# Biphasic regulation of the transcription factor ABORTED MICROSPORES (AMS) is essential for tapetum and pollen development in Arabidopsis

**DOI:** 10.1111/nph.14200

**Published:** 2016-10-27

**Authors:** Alison C. Ferguson, Simon Pearce, Leah R. Band, Caiyun Yang, Ivana Ferjentsikova, John King, Zheng Yuan, Dabing Zhang, Zoe A. Wilson

**Affiliations:** ^1^ Division of Plant & Crop SciencesSchool of BiosciencesUniversity of NottinghamSutton Bonington CampusLoughborough, LeicestershireLE12 5RDUK; ^2^ Faculty of BiologyUniversity of ManchesterMichael Smith Building, Oxford RoadManchesterM13 9PLUK; ^3^ School of MathematicsUniversity of ManchesterAlan Turing Building, Oxford RoadManchesterM13 9PLUK; ^4^ Centre for Plant Integrative BiologyUniversity of NottinghamSutton Bonington CampusLoughborough, LeicestershireLE12 5RDUK; ^5^ School of Mathematical SciencesUniversity of NottinghamNottinghamNG7 2RDUK; ^6^ Joint International Research Laboratory of Metabolic & Developmental SciencesShanghai Jiao Tong University–University of Adelaide Joint Centre for Agriculture and HealthSchool of Life Sciences and BiotechnologyShanghai Jiao Tong UniversityShanghai200240China

**Keywords:** aborted microspores (AMS), anther development, *Arabidopsis thaliana*, pollen development, regulatory network modelling, tapetum

## Abstract

- Viable pollen is essential for plant reproduction and crop yield. Its production requires coordinated expression at specific stages during anther development, involving early meiosis‐associated events and late pollen wall formation. The ABORTED MICROSPORES (AMS) transcription factor is a master regulator of sporopollenin biosynthesis, secretion and pollen wall formation in *Arabidopsis*. Here we show that it has complex regulation and additional essential roles earlier in pollen formation.
- An inducible‐AMS reporter was created for functional rescue, protein expression pattern analysis, and to distinguish between direct and indirect targets. Mathematical modelling was used to create regulatory networks based on wild‐type RNA and protein expression.
- Dual activity of AMS was defined by biphasic protein expression in anther tapetal cells, with an initial peak around pollen meiosis and then later during pollen wall development. Direct AMS‐regulated targets exhibit temporal regulation, indicating that additional factors are associated with their regulation.
- We demonstrate that AMS biphasic expression is essential for pollen development, and defines distinct functional activities during early and late pollen development. Mathematical modelling suggests that AMS may competitively form a protein complex with other tapetum‐expressed transcription factors, and that biphasic regulation is due to repression of upstream regulators and promotion of AMS protein degradation.

## Introduction

Anther development is a complex cascade of events regulating the differentiation of specialized cell types within the anther, involving > 1000 stamen‐specific transcripts in *Arabidopsis* (Alves‐Ferreira *et al*., 2007). The mature anther consists of four lobes, each containing meiotic cells at the centre surrounded by four somatic cell layers (the innermost tapetum, middle layer, endothecium and the outer epidermis) (Goldberg *et al*., 1993). Pollen development requires complex cooperative interactions between genes from the maternal (sporophytic) layers and gametophytic cells (Goldberg *et al*., 1993; Alonso *et al*., 2003; Wilson & Zhang, 2009; Xu *et al*., 2010). In particular, the tapetum surrounding the microspores plays a vital role in providing enzymes, nutrients and pollen wall components for the continued development of the pollen (Goldberg *et al*., 1993). In *Arabidopsis,* the tapetum is highly active between anther stages 5–9, then from stage 10 undergoes programmed cell death (PCD) to promote pollen development (Sanders *et al*., 1999; Parish & Li, 2010).

A number of principal tapetum transcription factors have been identified in *Arabidopsis,* including *DYSFUNCTIONAL TAPETUM1* (*DYT1*) (Zhang *et al*., 2006; Feng *et al*., 2012), *ABORTED MICROSPORES* (*AMS*) (Sorensen *et al*., 2003; Xu *et al*., 2010, 2014) (both basic helix‐loop‐helix (bHLH) transcription factors) and *MALE STERILITY1* (*MS1*) (a PHD finger‐motif transcription factor) (Wilson *et al*., 2001; Yang *et al*., 2007a). These are evolutionarily conserved, with orthologues characterized in rice and other cereals (Wilson & Zhang, 2009; Gómez *et al*., 2015). When mutated, they all result in pollen degeneration although they act at different stages. The *dyt1* mutant causes late meiotic failure during microspore development and prevents correct callose wall formation (Zhang *et al*., 2006; Feng *et al*., 2012); the *ams* mutant results in postmeiotic failure due to irregular tapetum development at anther stage 7, with microspore degeneration after the tetrad stage (Sorensen *et al*., 2003; Xu *et al*., 2014). In the *ms1* mutant there is failure after the single microspore stage, due to changed secretion and altered development of the tapetum (Vizcay‐Barrena & Wilson, 2006; Yang *et al*., 2007a).

A regulatory network based on mutant and interaction data has been suggested, with DYT1 upstream of both AMS and MS1 (Zhang *et al*., 2006), DYT1 regulating AMS through DEFECTIVE IN TAPETAL DEVELOPMENT AND FUNCTION 1 (TDF1) (Gu *et al*., 2014) and AMS regulating MS1 via MYB80 (Zhu *et al*., 2011; Feng *et al*., 2012; Lou *et al*., 2014). AMS has been shown to act as a master regulator of pollen wall development; the *ams* mutant shows failure of lipidic sporopollenin precursor accumulation (Xu *et al*., 2014) and extensive anther‐expressed genes (549) changes (Xu *et al*., 2010). Twenty‐three of these are confirmed as direct targets involved in sporopollenin biosynthesis and secretion, such as callose dissociation, lipidic transport, fatty acid elongation and phenolic compound formation (Xu *et al*., 2014).

In order to allow further insight into the transcriptional regulation of tapetum function we focused on defining the precise role and temporal activity of AMS during anther development. To probe this we created an inducible, functional AMS‐GR‐YFP fusion protein driven by its native promoter to analyse spatial and temporal expression in wild‐type and mutant backgrounds. Previously characterized AMS activities are associated with late events in pollen development (Xu *et al*., 2014), however here we reveal that it exhibits a complex regulatory expression and has a hitherto uncharacterized dual role during both early and late pollen development. We show that AMS function is facilitated by two peaks of protein expression within the tapetum, which are independent of transcriptional control. These peaks appear to be regulated independently and define essential, distinct roles at discrete stages of pollen and anther development. Mathematical modelling suggests that regulation is achieved by AMS competitively forming a protein complex, possibly with other tapetum‐expressed transcription factors, for example bHLHs 89 & 91 that are known to interact with AMS (Xu *et al*., 2010), and that the biphasic AMS expression could be created through the combined effects of MS1 indirectly promoting AMS protein degradation and repressing upstream TDF1 expression.

## Materials and Methods

### Plant materials and growth conditions

*Arabidopsis thaliana* (L.) Columbia‐0 (Col) and (*L*.) Landsberg *erecta*‐0 (L*er*) male sterile mutant lines were used to analyse AMS function in these different mutant backgrounds. These lines included the transcription factor SALK T‐DNA mutant lines *ams* (SALK_152147), *dyt1* (SALK_011257) and *myb26* (SALK_112372) (SALK SIGnal (Alonso *et al*., 2003); *ams* (Xu *et al*., 2010)*; myb26* (Yang *et al*., 2007b)), the X‐ray mutant line *male sterile35* (*ms35*) (Dawson *et al*., 1999) and the ethyl methanesulfonate (EMS) mutant line *ms1* (EMS (van der Veen & Wirtz, 1968); *ms1* (Wilson *et al*., 2001)). These mutant lines were sown in Levington M3 (The Scotts Company (UK) Ltd, Surrey, UK) compost supplemented with Met52 (Novozymes, Bagsvaerd, Denmark) and nematodes (Syngenta Bioline, Clacton‐on‐Sea, UK) and were grown in a glasshouse at 21°C : 17°C (day : night) and 22 h : 2 h photoperiod, along with their appropriate wild‐type (WT) control (ecotype Col). Plants were genotyped by PCR according to the specific papers referenced above using primers shown in Supporting Information Table S1.

### DEX inducible AMS construct

A 5‐kb region of *AMS* including a 3‐kb upstream region was amplified from genomic DNA of Col by PCR using primers AMSprom‐Kpn1_F1 and AMS_cDNA_AVRII_R1 (Table S1), and then cloned into TOPO PCR Blunt II (Invitrogen). The fragment was then digested with *Kpn*I and *Avr*II, and cloned upstream of (glucocorticoid receptor‐yellow fluorescent protein (GR‐YFP) in the pGREEN0229_PMYB26:MYB26‐GR‐YFP construct (C. Yang & Z. A. Wilson, unpublished) which had been digested with *Kpn*I/*Spe*I and the PMYB26:MYB26 replaced to create pGREEN0229_PAMS:AMS‐GR‐YFP. The construct was confirmed by PCR using primer pairs AMS_a_F/GFP_PGWB5_R and PG0229 F2/AMS PRO R4 (Table S1) then transferred into *Agrobacterium* (GV3101 + PSOUP) by electroporation (Sambrook *et al*., 1989). Arabidopsis heterozygous *ams* SALK T‐DNA line and Col plants were transformed by floral dipping (Clough & Bent, 1998). The T_1_ generation was screened for Basta^®^ resistance and PCR‐tested for the transgene. These plants grew to flowering stage, WT (Col) and the sterile plants with flower buds showing the *ams* mutant phenotype were dipped into 25 μM dexamethasone (DEX) + 0.02% Silwet L‐77 solution. YFP was observed using confocal microscopy (TCS SP2; Leica Microsystems (UK) Ltd, Milton Keynes, UK; 514 nm excitation wavelength, emission collection: 520–600 nm). DEX rescue experiments were based on five replicates from separate plants, which were treated either once, twice or three times, as appropriate; rescue subsequently was analysed across the whole inflorescence. pGREEN0229_PAMS:AMS‐GR‐YFP was crossed into male sterile backgrounds of *dyt1*,* ms1*,* myb26* and *ms35* and changes in YFP expression were observed. pGREEN0229_PAMS:AMS‐GR‐YFP was also crossed into the PMS1:MS1‐GFP Col background (Yang *et al*., 2007a), and YFP and green fluorescent protein (GFP) expression was observed (GFP: 488 nm excitation wavelength, emission collection: 495–510 nm).

### Expression analysis

Ribonucleic acid (RNA) was isolated from closed buds (RNeasy; Qiagen) and complementary DNA (cDNA) was prepared using 5 μg total RNA in a 20‐μl reaction (Superscript II reverse transcriptase; Invitrogen). Quantitative reverse transcriptase‐PCR (qRT‐PCR) was carried out using a Light Cycler (Roche) in a 384 plate set‐up. Reactions were set up using the Maxima SYBR Green QPCR Master Mix in a final volume of 9 μl, containing 0.2 μl of cDNA and 0.2 μl of the appropriate primers (Table S1). PCR cycling conditions for amplification were 95°C for 10 min, then 40 cycles of 95°C for 30 s, 58°C for 1 min and 72°C for 1 min. All samples were run at least in duplicate. Relative expression levels were determined in comparison to PP2A using the 2^ΔΔ^‐CT analysis method (Livak & Schmittgen, 2001).

### Microscopy

Pollen development progression was determined by staining isolated anthers using 2.0 μg μl^−1^ 4′,6‐diamidino‐2‐phenylindole (DAPI; Sigma) in aqueous solution (Tarnowski *et al*., 1991) after squashing and observation using a UV light microscope (Leica Microsystems (UK) Ltd). Pollen viability was assessed using Alexander cytoplasmic stain (Alexander, 1969) on isolated anthers; viable pollen stains purple whereas inviable stains green/clear. Fluorescence was detected using a Leica SP2 confocal laser scanning microscope (Leica Microsystems (UK) Ltd) (exciting fluorescence at 514 nm for YFP and 488 nm for chlorophyll auto fluorescence; YFP excitation collected at 520–600 nm; chlorophyll auto fluorescence collected at 660–700 nM; GFP exciting fluorescence at 488 nm with emission collection at 495–510 nm). Images were processed using the Leica SP2 Image Analysis software (Leica Microsystems (UK) Ltd), and further analysis on YFP and GFP intensity was done using Imaris ^™^ Spot recognition software (v.7; Bitplane, Oxford, UK). Two cross‐sections per anther were used for the fluorescence analysis and a minimum of eight anthers were used for each line per developmental point over two separate experiments. The whole developmental progression (inflorescence) was used to give a detailed observation of AMS‐YFP or MS1‐GFP over development, and DAPI staining was used to stage the material accurately. Staging of anthers is as described by Sanders *et al*. (1999).

### ChIP analysis

Chromatin immunoprecipitation (ChIP) analysis of AMS–DNA complexes was conducted as previously described using 1.5 g of whole closed Columbia buds (Xu *et al*., 2010). DNA from ChIP or input controls was purified using MinElute PCR Purification Kit (Qiagen). Real‐time PCR was performed as described in the Expression analysis section above and 0.2 μl DNA from ChIP/controls was used as a template. All samples were run at least in duplicate with at least two biological replicates. Quantification involved normalization of the cycle threshold (Ct) for each sample by subtracting the Ct of input control. Fold enrichment was calculated from Ct values by subtracting the Ct of the control (anti‐HIS) to obtain Δ Ct values, 2(ΔΔ Ct). Primers for qChIP‐PCR and electrophoretic mobility shift assay (EMSA) are shown in Table S1.

### EMSA analysis

The recombinant glutathione S‐transferase (GST)‐AMS protein was prepared as previously described using a GST pull‐down process (Xu *et al*., 2010). Deoxyribonucleic acid (DNA) fragments containing the E‐box of the regulatory region of target genes were labelled with digoxigenin (DIG) using the PCR DIG Probe Synthesis kit (Roche). Detection of the electrophoretic bands was performed by alkaline phosphatase conjugated anti‐digoxigenin antibody.

### Modelling

We constructed a set of mathematical models using ordinary differential equations to describe the mRNA and protein abundance of the key transcription factors (DYT1, TDF1, AMS, MYB80, MS1, bHLHs89/91). The initial model describes a cascade of genes, DYT1 – TDF1 – AMS – MYB80 – MS1 (see Methods S1). Potential transcriptional negative feedback loops (MS1 inhibiting itself, DYT1, TDF1 and AMS; AMS inhibiting DYT1 and TDF1) were included in the models, as shown later in Fig. 4a, as was the assumption that MS1 protein increases the rate at which AMS protein is degraded. These models were built on, for example by the addition of bHLH interacting proteins to induce downstream activation, to give the final model (see later Methods S1; Fig. 4). In the final model, TDF1 is activated by DYT1, in a complex with the bHLHs, and activates AMS. AMS forms a complex with the bHLHs to activate MYB80, which activates MS1. MS1 increases the rate at which the AMS protein is degraded (possibly indirectly through another gene, the precise mechanism is not included here), without acting on the mRNA levels. The majority of the transcriptional negative feedback loops were rejected as nonsignificant in the model selection process, leaving only MS1 inhibiting TDF1 and AMS inhibiting DYT1 (see later Fig. 4c).

The expression of DYT1 and the additional bHLHs are assumed to be driven by genes that are further upstream and hence outside of the scope of this work, and therefore their mRNA production rates are fitted by exponential distributions. For specificity here, we used the sum of bHLH89 and bHLH91 as the interacting bHLH levels, although other transcription factors may be involved.

The predicted mRNA levels from the model were fitted to qRT‐PCR expression from staged material from Col‐0 plants, and the AMS and MS1 protein concentrations were fitted to the mean values measured using Imaris software (Table S2) (v7; Bitplane). These nine samples were used as a proxy for time and correspond to the following anther stages: 1, pre‐meiotic PMC (anther stage 5); 2, meiosis (anther stage 6); 3, tetrad (anther stage 7); 4, free microspores (anther stage 8); 5, mitosis (anther stages 9, 10); 6, 7: bicellular (anther stage 11); 8, tricellular (anther stage 12); and 9, mature pollen (anther stage 12). To perform the fitting, we used the genetic algorithm provided in Matlab (release 2014a; The MathWorks Inc., Cambridge, UK) to find a parameter set that minimizes the weighted squared distance to the experimental data. The final best‐fit values of the parameters are given in Methods S1. The robustness of this fit was analysed using the Hyperspace algorithm (Zamora‐Sillero *et al*., 2011), showing that the majority of the parameter estimates are reasonably well constrained (Methods S1).

### Microarray re‐analysis

As previously described (Pearce *et al*., 2015), the two‐colour microarray data of (Xu *et al*., 2010), comparing WT and *ams* buds, was re‐normalized using the Bioconductor ‘limma’ package (Smyth, 2005) in the programming language R, using the ‘normexp’ background correction method, followed by within‐array median normalization and between‐array quantile normalization. Genes are considered differentially expressed in a specific sample if their average expression (*A*) is over 6 (on a log^2^ scale), and they are at least two‐fold differentially regulated (|*M*| > 1) in the same direction (up or down) in all three individual replicates of a sample. This criteria gives 224, 335, 430, 449 genes as being differentially expressed in the *ams* mutant for the meiosis, mitosis I, bicellular and mitosis II stages, respectively. As some genes are differentially expressed in multiple stages, this gives 980 genes in total.

### Accession numbers

AMS (At2g16910), MS1 (At5g22260), DYT1 (At4g21330), TDF1 (At3g28470), MYB26 (At3g13890), bHLH89 (At1g06170), bHLH91 (At2g31210), bHLH10 (At2g31220), MYB80 (At5g56110).

## Results

### Dexamethasone (DEX)‐inducible expression of AMS rescues the *ams* mutant

**Figure 1:**
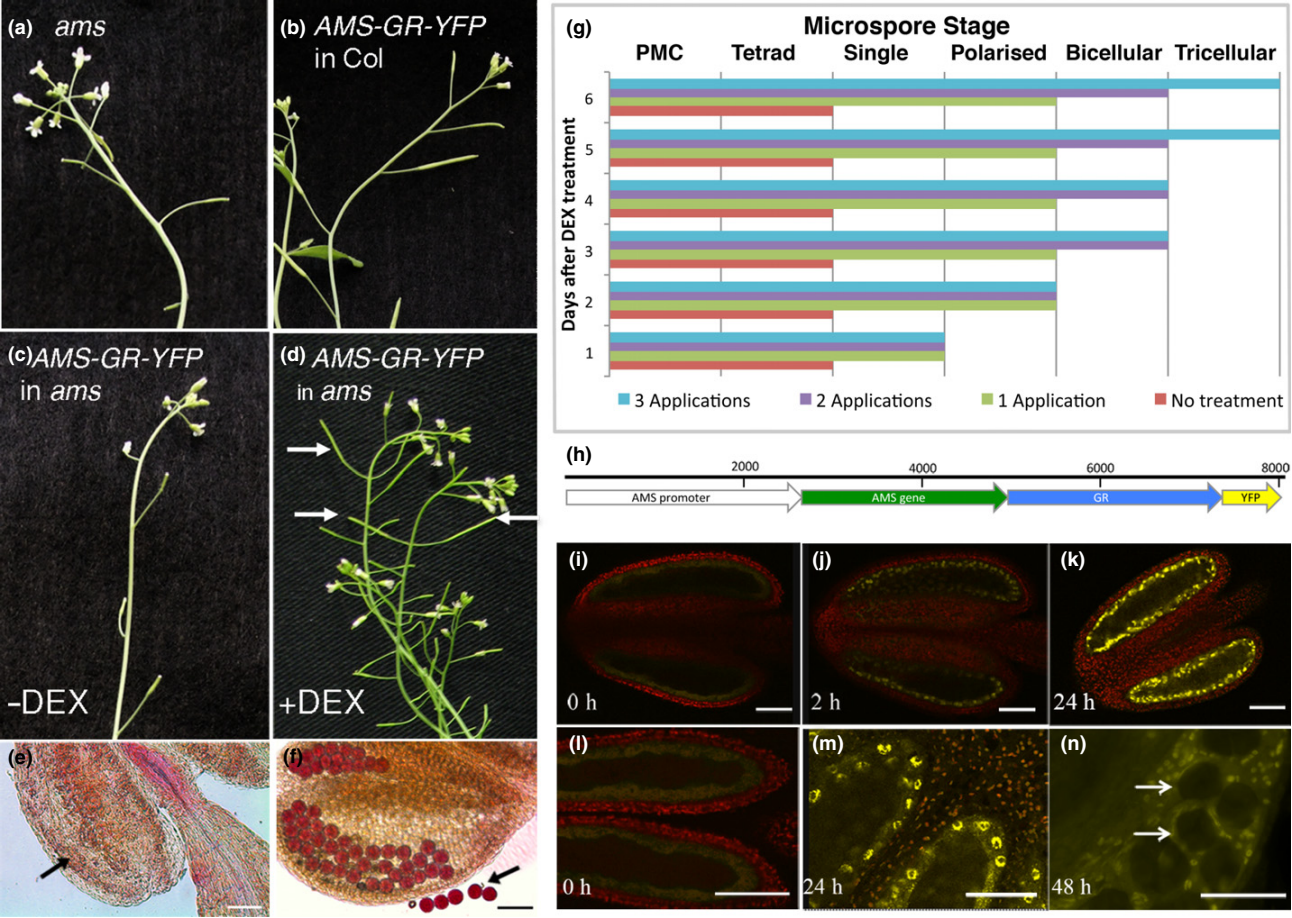
Rescue of pollen development and fertility in the *ams* mutant by dexamethasone (DEX)‐induced expression of ABORTED MICROSPORES (AMS). (a–d) Silique development in the *Arabidopsis thaliana* (a) *ams* mutant, (b) PG228_PAMS:AMS‐GR‐YFP in Col background, (c) PG228_PAMS:AMS‐GR‐YFP in *ams* background without DEX treatment showing *ams* mutant phenotype, and (d) PG228_PAMS:AMS‐GR‐YFP in *ams* background with DEX treatment showing rescue of fertility (arrows). (e, f) Alexander staining of anthers from PG228_PAMS:AMS‐GR‐YFP in *ams* background, (e) without DEX treatment showing degenerative inviable pollen (arrow), and (f) with DEX treatment showing viable pollen stained purple (arrow). (g) Progress of rescue of pollen development after DEX induced expression of PG228_PAMS:AMS‐GR‐YFP in *ams* mutant when treated with one application of DEX (day 0), two applications of DEX (days 0 and 2), and three applications of DEX (days 0, 2 and 4) compared to no treatment; data are representatives of five replicates. Pollen development is halted prematurely unless DEX treatment is maintained for ≥ 5 d, indicating a requirement for functional AMS across multiple developmental stages from the tetrad to bicellular stages. (h) PG228_PAMS:AMS‐GR‐YFP construct. (i–l) Confocal analysis of anthers from the *ams* mutant containing the PG228_PAMS:AMS‐GR‐YFP construct. (i, l) Cytoplasmic YFP expression before DEX treatment, (j) nuclear YFP expression 2 h post‐DEX treatment, (k, m) nuclear YFP expression 24 h post‐DEX treatment. (n) Squashed anther showing AMS‐YFP tapetum specific localization, arrows show tetrad microspores with no YFP expression 48 h post‐DEX treatment. Bars: (e, f) 50 μm; (i–n) 12.5 μm.

A translational AMS inducible‐fusion protein under the native AMS promoter (PAMS:AMS‐GR‐YFP) was transformed into heterozygous *amsAMS* mutant plants; transformed sterile plants segregated (3 : 1) for fertility and were confirmed by PCR as *ams* mutants and as carrying the transgene. These plants exhibited the *ams* male sterile phenotype and showed AMS‐GR‐YFP expression in the tapetum cytoplasm due to the glucocorticoid receptor (GR) ligand‐binding domain (Fig. 1i,l), therefore this was unable to rescue fertility. DEX‐treatment resulted in nuclear localization of the AMS protein for a 36–48 h period (Fig. 1j,k,m,n); three DEX treatments (days 1, 3 and 5) were able to rescue fertility fully, after which the plants reverted back to sterility unless DEX‐treatments were maintained. Flowers from WT, or *ams‐*rescued lines, carrying the transgene appeared normal. Although all of the inflorescences from the plant were treated with DEX (therefore all bud stages), rescue depended on anther stage (Fig. 1g); buds containing postmeiotic pollen (anther stage 7 onwards) were sterile, whereas before pollen mother cell (PMC) meiosis (anther stages 5, 6), and in buds developing during the treatment, full fertility occurred with normal pollen development and silique elongation. Progression of anther development across whole inflorescences were observed for 6 d in four experiments, treated with DEX either once (day 0), twice (days 0 and 2), three times (days 0, 2 and 4) and an untreated control. Full rescue required multiple DEX applications; a single treatment only enabled development from microspore mother cell (anther stage 5) to microspore/polarized microspore stage (anther stage 8) (Fig. 1g). This block in developmental progression indicates that nuclear‐localized AMS is required at multiple stages in the anther tapetum.

**Figure 2:**
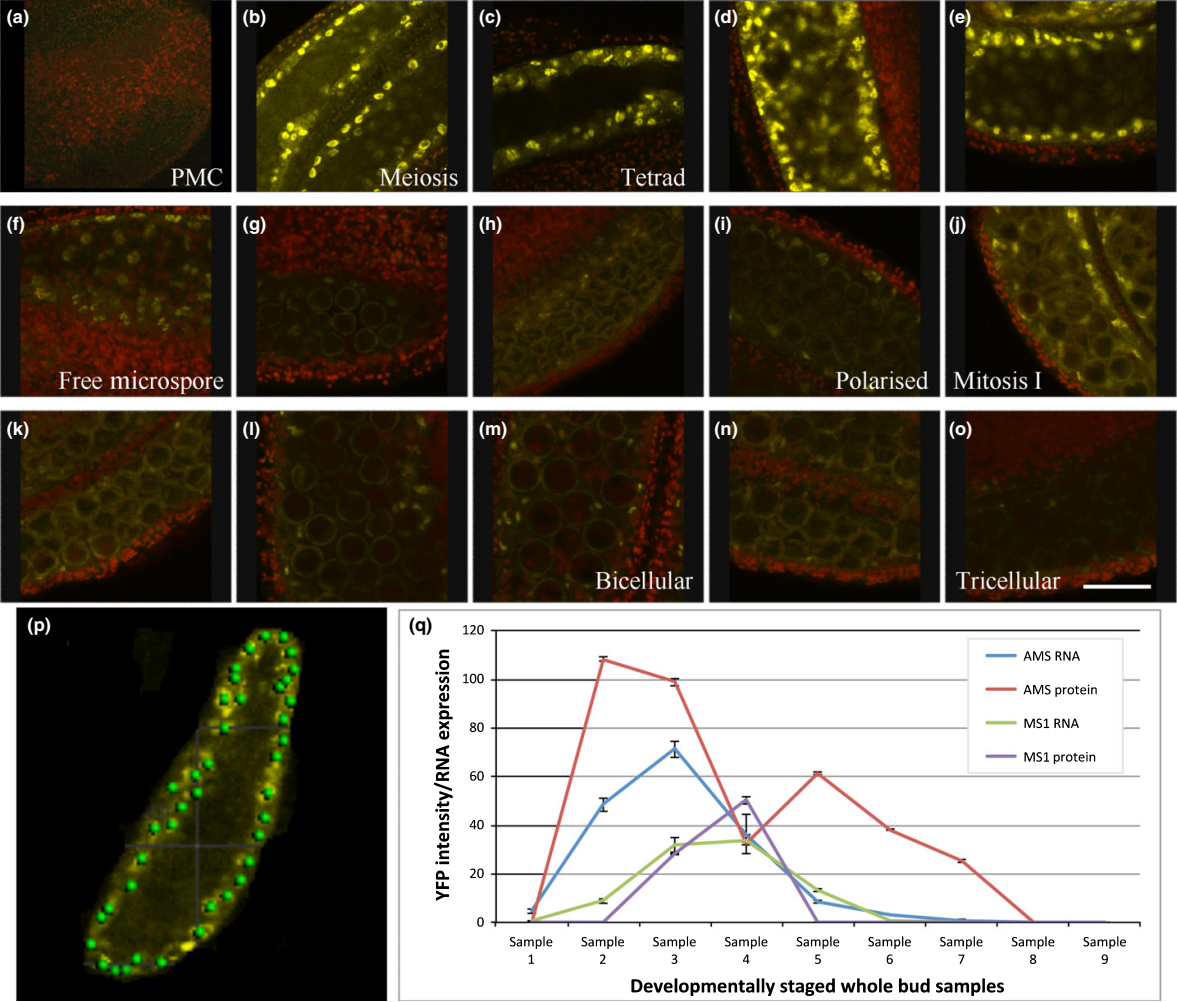
Expression levels of ABORTED MICROSPORES (AMS) and MS1 protein during pollen development. (a–o) Staged AMS‐YFP expression in PG228 pAMS::AMS‐GR‐YFP in *Arabidopsis thaliana* Col background 24 h after dexamethasone (DEX) treatment. (a) Pollen mother cell (PMC) – pre‐meiosis (anther stage 5), (b) PMC – meiosis (anther stage 6), (c) PMC – meiosis (anther stage 6), (d, e) tetrad (anther stage 7), (f–h) single free microspore (anther stage 8), (i) polarized microspore (anther stage 9), (j–l) mitosis I (anther stage 10), (m, n) bicellular (anther stage 11), (o) tricellular (anther stage 12). Representative image of 16 anthers over four experimental replicates for each developmental stage. Bar, 20 μm. (p) Imaris spot recognition software used to measure AMS‐YFP and MS1‐GFP intensity in the nucleus. (q) *AMS* and *MS1* RNA levels (quantitative reverse transcription polymerase chain reaction, qRT‐PCR) in wild‐type (WT) (Col) in comparison to AMS protein (YFP intensity) and MS1 protein (GFP intensity) in Col background containing PG228 pAMS::AMS‐GR‐YFP and pMS1::MS1‐GFP construct 24 h after DEX addition. Samples: (1) pre‐meiotic PMC (anther stage 5); (2) meiosis (anther stage 6); (3) tetrad (anther stage 7); (4) free microspores (anther stage 8); (5) mitosis I (anther stages 9, 10); (6, 7) bicellular (anther stage 11); (8) tricellular (anther stage 12); (9) mature pollen (anther stage 12). Data are representative of means of at least 12 anthers across three replicates ± SE.

Before DEX‐treatment, the AMS‐YFP protein was observed in the tapetum cytoplasm; nuclear localization was seen within 2 h of DEX application to dissected anthers (Fig. 1j), or 4 h when applied to the whole plant. Strong nuclear expression was seen *in planta* 12–48 h post‐DEX treatment, in the tapetum but not microspores (Fig. 1n); 48 h post‐DEX treatment cytoplasmic expression was also detected and by 72 h AMS‐YFP was solely cytoplasmically localized. Multiple WT lines carrying the PAMS:AMS‐GR‐YFP fusion protein were analysed using complete inflorescence developmental series for localization of the AMS‐YFP protein; staging was confirmed by DAPI analysis (Fig. S1a–o corresponds to Fig. 2a–o). Two distinct peaks of high‐concentration AMS‐YFP protein were seen at: pollen meiosis to tetrad stage (Fig. 2b–e; anther stages 6, 7); and pollen mitosis I (Fig. 2j; anther stage 10); whereas staged qRT‐PCR indicated a single peak of prolonged *AMS* expression (Fig. 2q). Weak YFP expression was observed between free microspore to bicellular pollen (Fig. 2f–i, k–n; anther stages 8–11); before pollen meiosis (anther stage 5) and after pollen mitosis II (anther stage 12) no AMS‐YFP expression was detectable (Fig. 2a,o).

### AMS protein expression is inversely correlated with MS1 protein expression

**Figure 3:**
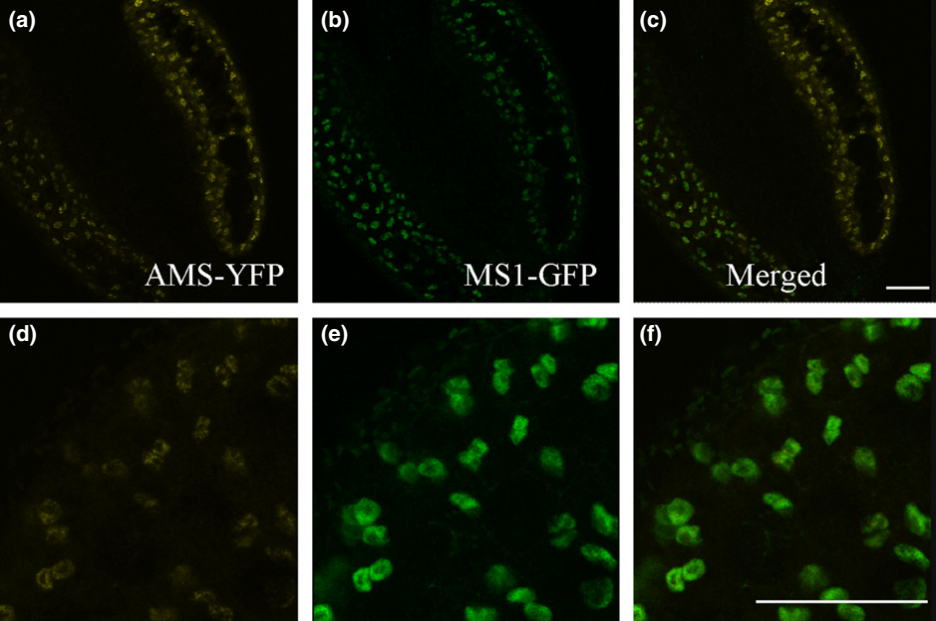
AMS‐YFP and MS1‐GFP are coexpressed in the nuclei of tapetum cells at late tetrad stage. (a, d) AMS‐YFP, (b, e) MS1‐GFP, and (c, f) merged image, in PG228_PAMS:AMS‐GR‐YFP crossed with PMS1:MS1‐GFP in *Arabidopsis thaliana* Col background, 24 h after dexamethasone (DEX) treatment. Representative image of eight anthers over two experimental replicates (see also Supporting Information Fig. S3). Bars, 10 μm.

It has been previously proposed that the PHD‐finger transcription factor MS1 functions downstream of AMS (Feng *et al*., 2012). We generated an *ams ms1* double mutant, which presented the *ams* phenotype (Fig. S2c,f). We also introgressed our functional tapetum‐specific MS1‐GFP translational fusion construct (PMS1:MS1‐GFP) (Yang *et al*., 2007a) into the *ams* mutant; no MS1‐GFP expression was seen, confirming AMS acts upstream of MS1 (Fig. S2h). The PAMS:AMS‐GR‐YFP construct was also introgressed into the MS1‐GFP translational fusion line (Yang *et al*., 2007a). AMS‐YFP and MS1‐GFP were observed specifically in tapetum nuclei in the WT background after DEX treatment. Concentrations of MS1 protein correlated negatively with *AMS* RNA and protein expression, with a tight link between upregulation of MS1‐GFP protein and reduction in AMS‐YFP protein (Figs 2q, S3). AMS‐YFP and MS1‐GFP were observed specifically in the nuclei of the tapetum cells, with co‐expression of the two proteins occurring as the microspores developed from tetrad to single microspore stage (Fig. 3). At the tetrad stage (anther stage 7), high AMS‐YFP was seen, with low MS1‐GFP; after this, at microspore release from the tetrads, AMS‐YFP expression decreased while MS1‐GFP increased. At the free single microspore stage (anther stage 8) a reversal of expression levels were seen, with high MS1‐GFP and low AMS‐YFP (Fig. S3). This suggests an inverse correlation between the protein concentrations for these transcription factors.

### Mathematical modelling predicts biphasic AMS is regulated by competitive transcription factor binding and MS1‐mediated degradation

**Figure 4:**
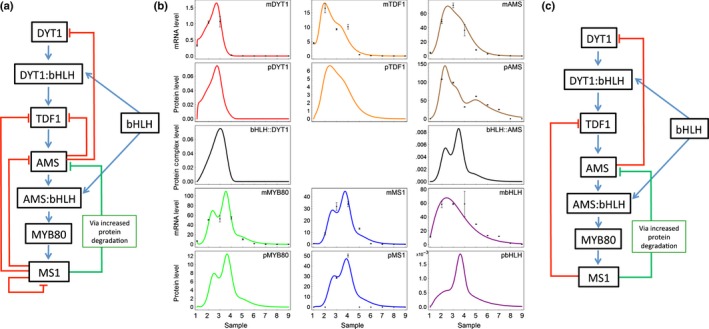
Mathematical modelling of ABORTED MICROSPORES (AMS) network in *Arabidopsis*. (a) Proposed model of the AMS network, including the formation of complexes between the bHLHs, DYT1 and AMS (represented by colons). (b) Output of the mathematical model, showing the prediction of the best fit of the model (lines), with the measured protein and mRNA levels shown as points. Samples: (1) pre‐meiotic PMC (anther stage 5); (2) meiosis (anther stage 6); (3) tetrad (anther stage 7); (4) free microspores (anther stage 8); (5) mitosis I (anther stages 9, 10); (6, 7) bicellular (anther stage 11); (8) tricellular (anther stage 12); (9) mature pollen (anther stage 12). Error bars show ± SE, protein values are only available for AMS and MS1 (see Methods S1 for model details). (c) Final regulatory network as generated from mathematical modelling, after discarding negative feedbacks with low weight.

We constructed a mathematical model to investigate the control of AMS expression, with a cascade of DYT1, TDF1, AMS, MYB80 and MS1 transcription factors and potential negative feedbacks between them, as suggested by the expression data and published network interactions (Fig. 4a; Methods S1). DYT1 transcription dynamics were assumed to take a Gaussian form (regulated by upstream factors not included in the model). The messenger RNA (mRNA) and protein concentrations were described by ordinary differential equations that depend on (unknown) rates of transcription, translation and degradation. In order to de‐couple the AMS transcript and protein concentrations and capture the second AMS protein peak, we included a term representing the enhanced degradation of AMS protein by MS1. This is likely to be occurring through intermediaries, although the exact mechanism remains to be determined.

Our initial models (Methods S1) were unable to fit the predicted dynamics to transcript levels measured by qRT‐PCR in staged WT material and the measured AMS and MS1 protein concentrations (Fig. 2q). To generate a better fit to the data, we included additional terms to represent complexes forming with DYT1 and AMS to alter their activity, as previous data (Xu *et al*., 2010; Feng *et al*., 2012; Cui *et al*., 2016), suggests DYT1 and AMS interact with a group of bHLH transcription factors (bHLHs 89/91). These bHLHs act redundantly, dimerise *in vitro* and form complexes with DYT1 and AMS (Xu *et al*., 2010; Feng *et al*., 2012; Zhu *et al*., 2015; Cui *et al*., 2016), and may promote transcription of downstream network components (TDF1 (Cui *et al*., 2016) and MYB80, respectively). Incorporation of competitive binding between DYT1, AMS and the bHLHs enabled a good fit to the measured transcript and protein dynamics (Fig. 4b). Performing a global parameter sensitivity analysis revealed that a minimal model captured the observed dynamics (Fig. 4c), in which bHLH complex formation and MS1‐promoting AMS protein degradation were essential to create the two‐peaked AMS dynamics. The model demonstrates how the initial increase in DYT1 causes an increase in all downstream components; however, the fitted model parameters are such that the bHLHs associate more rapidly with DYT1 than with AMS, delaying significant production of MYB80 and MS1 until DYT1 concentrations drop at tetrad stage (sample 3; anther stage 7), enhanced by AMS inhibiting DYT1 itself. Later, rising MS1 concentrations promote rapid AMS protein degradation, resulting in lowered AMS protein concentrations at free microspore stage (sample 4; anther stage 8), despite AMS transcript still being present. This decrease of AMS protein in turn reduces MS1 protein synthesis, removing the promotion of AMS degradation and causing a short‐lived second peak in AMS protein concentrations at mitosis I (sample 5; anther stages 9, 10). Finally, negative feedback of TDF1 transcription by MS1 (along with the lack of DYT1 to promote TDF1) causes all the mRNA and protein concentrations to reduce to zero. As described below the network model was validated using data from both the *ms1* and *ams* mutants (Methods S1 Section 5 – Simulation of mutant phenotypes), and shows that the model successfully predicts the qualitative behaviour observed in the mutants for AMS mRNA and protein expression as determined by qRT‐PCR and confocal microscopy of the PAMS:AMS‐GR‐YFP fusion protein in male sterile mutant backgrounds.

### AMS‐YFP protein expression is prolonged in male sterile mutants

**Figure 5:**
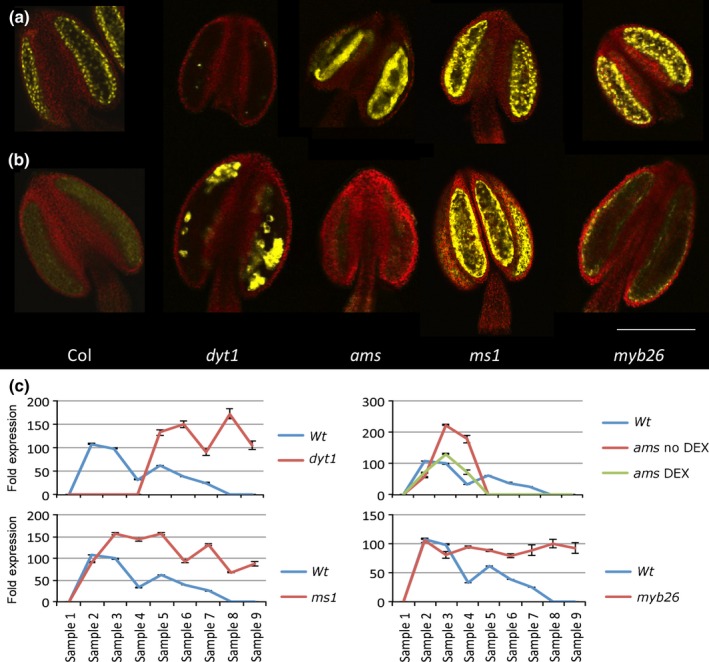
AMS‐YFP expression differs in different male sterile mutant backgrounds. Confocal analysis of AMS‐YFP expression of PG228_PAMS:AMS‐GR‐YFP in the *Arabidopsis thaliana* male sterile mutant backgrounds, *dyt1, ams, ms1* and *myb26* at (a) tetrad (anther stage 7) and (b) single microspore (anther stage 8). Representative image of at least eight anthers over two experimental replicates. Bar, 50 μm. (c) Imaris software analysis of YFP intensity in the different mutant backgrounds compared to the Col (wild‐type, WT) background 24 h post‐dexamethasone (DEX) treatment. The PG228_PAMS:AMS‐GR‐YFP in the *ams* mutant background has both no DEX and 24 h post‐DEX to compare expression patterns. Samples: (1) pre‐meiotic PMC (anther stage 5); (2) meiosis (anther stage 6); (3) tetrad (anther stage 7); (4) free microspores (anther stage 8); (5) mitosis I (anther stages 9, 10); (6, 7) bicellular (anther stage 11); (8) tricellular (anther stage 12); (9) mature pollen (anther stage 12). Data are representative of means ± SE.

AMS‐YFP dynamics were analysed in the *ams, dyt1*,* ms1* and *myb26* (also known as *male sterile35 (ms35)*) mutants*,* by experimental and modelling systems. The inducible AMS construct was introgressed into these different male sterile mutants and staged material was observed for AMS‐YFP protein expression and transcriptional changes. In the *ams* mutant, higher AMS‐YFP protein was detected during early anther development (Fig. 5c), consistent with model predictions (Methods S1) the observed expression peaked (anther stages 6, 7) and then faded completely. This corresponds to tetrads and tapetum degeneration within the mutant, in contrast to WT where weak expression was observed from anther stage 8 onwards followed by the second peak at stage 11 (Fig. 5c). The heightened fluorescence in *ams* was reduced 24 h post‐DEX treatment, indicating that functional, nuclear‐localized AMS directly or indirectly downregulates its own expression, possibly via negative regulation of an upstream positive regulator (e.g. DYT1), or a downstream negative regulator (e.g. MS1).

DYT1 regulates *AMS* expression indirectly via the *TDF1/MYB35* transcription factor (Feng *et al*., 2012; Gu *et al*., 2014). This is supported by our lack of AMS‐YFP protein expression in early buds in the *dyt1* mutant. Surprisingly in *dyt1*, patches of high‐intensity AMS‐YFP were detected later in tapetum development and were maintained throughout the remaining anther stages (Fig. 5b), this was also observed in the expression analysis with late high upregulation (Fig. S4a). This suggests that additional factor(s), other than DYT1, are regulating AMS expression during late anther development and pollen wall formation, possibly via alternative regulation of TDF1 (which also shows a delayed upregulation in qRT‐PCR analysis; Fig. S4c). Recent research showed that *AMS* expression is restored in *dyt1* mutant expressing proDYT1::TDF1 construct (Gu *et al*., 2014).

In the *ms1* mutant, early AMS‐YFP protein appeared normal until anther stage 7, however, unlike WT, there was no decrease with AMS protein and transcript remaining high as the tapetum degraded (Figs 5d, S4a). This suggests that MS1 is involved, directly or indirectly, in negatively regulating *AMS* expression, which is consistent with our model, where MS1‐inhibiting TDF1 transcription is required to reproduce the WT dynamics (Methods S1). Furthermore, upregulation of *AMS* expression was observed in our re‐analysis of *ms1* microarrays (Pearce *et al*., 2015).

Unlike the previous mutants, *myb26* produces viable pollen but has later defects associated with anther dehiscence. As in *ms1*, early AMS protein in *myb26* was normal but concentrations subsequently plateaued to lower than WT and was visible in isolated cells until the final stages of tapetum breakdown, and RNA transcript stayed expressed (Figs 5b,c, S4). This suggests that MYB26 may have a role in negatively modulating *AMS* expression, albeit at later stages of pollen development.

### AMS binds directly to MYB80, DYT1 and TDF1

**Figure 6:**
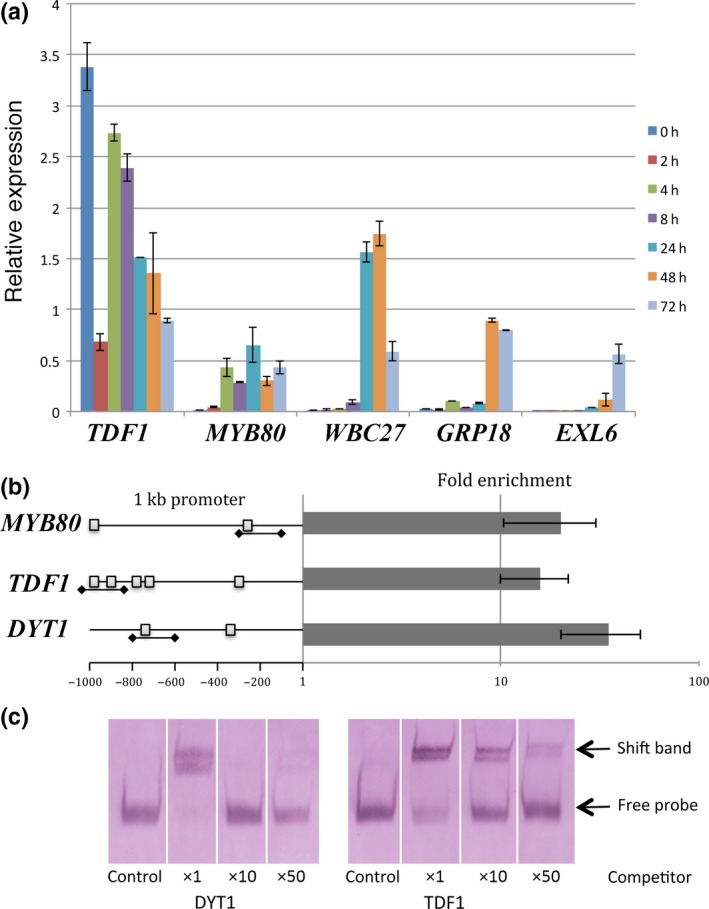
Differing gene induction of ABORTED MICROSPORES (AMS) direct targets over a DEX inducible time‐course and confirmation of direct interaction for DYT1 and TDF1. (a) Gene expression of exemplar AMS direct targets showing down‐ or upregulation over the 4 d time‐course in PG228_PAMS:AMS‐GR‐YFP in *Arabidopsis thaliana ams* background. Each gene has been normalized to expression at 0 h dexamethasone (DEX) addition in PG228_PAMS:AMS‐GR‐YFP in Col background. Data are representative as means ± SE. (See also Fig. S5.) (b) qChIP‐PCR analysis of the enrichment of AMS upstream targets, the downstream target *MYB80* was used as a positive control. Fold enrichment calculations from qPCR assays in two independent ChIP experiments, calculated by fold change between the ChIP (anti‐AMS) and control (anti‐HIS) experiment. Data are representative of means ± SE. The predicted E‐boxes within the promoter regions are indicated as small squares, and the PCR probes used for ChIP and electrophoretic mobility shift assay (EMSA) are underlined. (c) EMSA using digoxigenin labelled probes were observed without AMS protein or unlabelled competitor probes (control) and with AMS protein in the presence of ×1, ×10 and ×50 unlabelled competitor probes.

AMS has been shown to bind to the ‘E‐box’ motif (CANNTG) (Xu *et al*., 2010); direct binding to the MYB80 promoter has also been recently demonstrated (Lou *et al*., 2014) and is supported by our inducible AMS data where it is an early direct target (Fig. S5). In the absence of functional AMS protein there is upregulation of *DYT1* and *TDF1* expression (Fig. S4b,c). EMSA and qChIP‐PCR analysis revealed that AMS can directly bind to an E‐box in the region upstream of DYT1 and TDF1 (Fig. 6b,c). This indicates that AMS plays a direct role in regulating these factors and that there is tight control of the network via activation and subsequent repression, via feedback loops (Fig. 4a). Nevertheless our modelling indicates that although AMS directly regulates DYT1 expression, TDF1 regulation is primarily via MS1 inhibition (Fig. 4c), with direct repression via AMS playing a minor role.

### Direct AMS targets require additional factors for induction

A number of AMS downstream targets were tested by qRT‐PCR for induction over a 96‐h time‐course using the PAMS:AMS‐GR‐YFP construct in the *ams* mutant (daily DEX‐treatment to maintain nuclear localization and compared directly to DEX‐treated *ams* as a control). Most of the known direct AMS targets (Xu *et al*., 2010, 2014) were not induced in the 2–8 h post‐DEX treatment that is expected of primary regulatory targets and many were induced 24–72 h post‐DEX treatment (Figs 6a, S5). The timing of induction corresponded closely with WT expression pattern; genes expressed early in tapetal development were induced early during the time‐course (e.g. MYB80), whereas those expressed later during pollen wall formation (e.g. EXL6) were induced later. This indicates additional regulatory control exists to differentially regulate genes in a developmental order, regardless of the presence of functional AMS protein, suggesting that other factor(s) may be involved in reinforcing expression to create this time delay.

## Discussion

**Figure 7:**
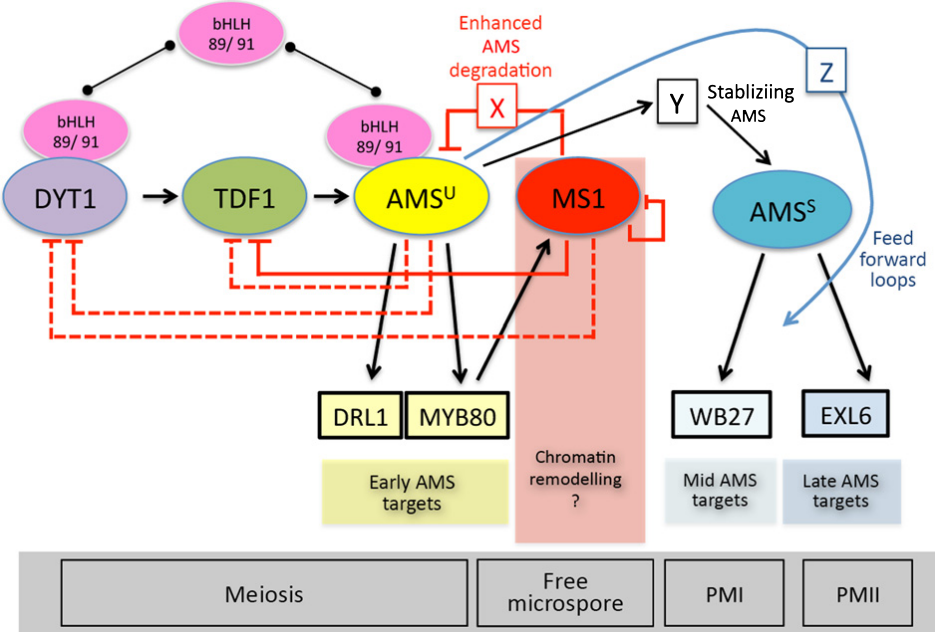
A proposed network for the regulation of tapetum transcription factors during anther development in *Arabidopsis thaliana*. The ABORTED MICROSPORES (AMS) protein exhibits a biphasic expression pattern in the anther tapetum, with distinct regulatory targets and defined functional activities during early and late anther development. Control of this regulation may be achieved via feed‐forward loops. Modelling predicts a competitive interaction between AMS and other tapetum‐expressed transcription factors, potentially the bHLH89 and bHLH91 transcription factors, and DYT1. It is proposed that the two AMS protein peaks could be created through the combined effects of the MS1 transcription factor indirectly promoting AMS protein degradation and by repression of upstream regulators. AMS^U^, unstable protein; AMS^S^, stable protein; X, Y and Z, unknown factors; arrows, direct regulation; lines ending with a line, repression; lines with a line ending with circle, protein interactions. Dashed lines indicate a minor role in regulation of network (as predicted by modelling).

Functional aborted microspores (AMS)‐yellow fluorescent protein (YFP) protein shows two peaks of high expression in the anther tapetum nuclei between pollen meiosis to tetrad stages (anther stages 6, 7), then later during pollen mitosis I (anther stage 10); whereas although *AMS* transcript encompasses these stages the transcript expression peaks at tetrad stage (anther stage 7) and then gradually declines (Fig. 2). The timing of the second AMS peak is consistent with the role for AMS as a master regulator of pollen exine formation (Xu *et al*., 2014). However, the first peak occurs earlier, coinciding with late meiosis and tapetum programmed cell death (PCD) initiation, suggesting a dual role for AMS during anther development (Fig. 7). Consistent with this, dexamethasone (DEX)‐induction confirmed that AMS is required at multiple stages (Fig. 1g), with anther/pollen developmental stage being critical for rescue. Only pre‐meiotic stages (anther stages 5, 6) can be rescued with DEX treatment, however, full rescue requires multiple applications, with one application only rescuing at the tetrad stage to allow formation of single microspores. Additionally there is associated temporal regulation of AMS‐direct targets (Fig. 6a), suggesting that this requirement for AMS indicates multiple distinct roles for the AMS protein during pollen formation. AMS expression appears regulated at multiple levels; increased nonfunctional AMS‐YFP‐ glucocorticoid receptor (GR) (protein and transcript) is seen in the *ams* mutant introgressed with the inducible construct (Fig. 5c), implying a self‐regulatory feedback loop, possibly via MYB80 or MS1. Both of these genes are downregulated in the *ams* mutant, whereas *AMS* is upregulated in *myb80* and *ms1* (Alves‐Ferreira *et al*., 2007; Phan *et al*., 2011), and AMS‐YFP in *ms1* (Figs 5c, S4). Alternatively, negative regulation of upstream regulators (e.g. DYSFUNCTIONAL TAPETUM1 (DYT1) or DEFECTIVE IN TAPETAL DEVELOPMENT AND FUNCTION 1 (TDF1)) would allow for the observed higher AMS protein and RNA expression. In the model, the two peaks of high expression of MYB80 and MS1 match the two peaks in bHLH:AMS binding, and reflect the change of bHLH89/91 binding to DYT1 and AMS, to a shift of binding to AMS solely. This may be due to the oversimplification of the model by combining the two bHLHs expression data together, because these two proteins may show differential binding between the two proteins AMS and DYT1, which would allow the protein complex to be a smoother curve. As we only have data at distinct points, the fitting function of the model can affect values between these points, and the two peaks in bHLH:AMS, MYB80 and MS1 may be an artefact of the fitting process. This will be addressed in future work to further expand and confirm the model. Our data indicate that AMS negatively directly regulates DYT1 and TDF1, possibly allowing a negative feedback loop to the early transcriptional regulators (Figs 6, S4). Our modelling predicts that the network can be generated predominantly via AMS inhibiting DYT1 and MS1‐mediated repression of TDF1, although the full impact of AMS on downregulating DYT1 is slightly unclear because the model does not include the genes upstream of DYT1. This negative feedback occurs within 24 h, causing AMS‐YFP to return to wild‐type (WT) concentrations after DEX induction (Fig. 5c).

The WT drop in AMS‐YFP after the first peak (tetrad stage; anther stage 7) correlates with increased MS1‐GFP protein, suggesting close regulation of AMS by MS1 (Fig. 2q). The MS1 protein is extremely transient (Yang *et al*., 2007a) and is not present during the second AMS peak; this transient expression appears important for AMS downregulation and network dynamics.

Translational or post‐translational regulation of AMS is suggested by the lack of correlation between AMS protein and RNA expression (Fig. 2q). AMS could initially be rapidly turned over while RNA expression is high, whilst the later AMS peak may reflect the protein becoming stabilized, or inhibition of protein turnover, allowing concentrations to increase despite reduced RNA levels (Fig. 7). Our model supports this by assuming that MS1 increases AMS protein degradation; this predicts the second AMS protein peak due to the reduction in AMS degradation as MS1 declines. In the *ms1* mutant, during the early stages of anther development the concentrations of AMS‐YFP appear normal; however, in the later stages, the normal decreased expression of the AMS protein is not seen; suggesting that MS1 negatively regulates *AMS* expression, possibly through chromatin remodelling, but also via indirect protein degradation as proposed by our modelling. This agrees with the inverse correlation of MS1‐GFP and AMS‐YFP (Figs 2q, S3) and correctly predicts the increased AMS protein in the *ms1* mutant (Fig. 5c). *AMS* mRNA concentrations in the *ams* mutant (Fig. S4) are upregulated without declining after anther stage 7 yet the AMS‐YFP protein disappears at stage 7; suggesting that a downstream factor may also be involved in stabilizing AMS protein, allowing the second protein peak.

In the *ams* mutant 980 genes are two‐fold differently regulated, including 69 transcription factors (reanalysis of microarray data (Xu *et al*., 2010); Table S3), suggesting that AMS may regulate tapetal development via additional transcription factors. AMS directly regulates 23 targets associated with pollen wall biosynthesis (Xu *et al*., 2010, 2014); nevertheless these targets show temporal specificity (Figs 6a, S5). Some (e.g. *MYB80* and *DEFORMED ROOTS AND LEAVES* (*DRL1*)) were induced by 4 h, whilst others (e.g. *LAP5, WBC27* and *CYTOCHROME P450 703A2* (*CYP703A2*)) by 24, 48 (e.g. *GLYCINE‐RICH PROTEIN 18* (*GRP18*)*, LIPID TRANSFER PROTEIN 12* (*LTP12*) and *3‐KETOACYL‐CO‐A SYNTHASE 7* (*KCS7*)) or 72 h (*QUARTET 3* (*QRT3*)*, GRP19* and *EXORDIUM LIKE 6* (*EXL6*)). Genes expressed early in WT, such as *DRL1* (stages 4–9; Grienenberger *et al*., 2010), were induced rapidly. Later targets, for example *WBC27* (induced 24 h after AMS induction), are normally expressed from Anther stages 7–11 (Dou *et al*., 2011), whilst *EXL6,* which is expressed in WT from anther stage 9 (Xu *et al*., 2014) and was induced 72 h after DEX induction of AMS. This suggests that AMS is not solely responsible for their activation, but additional mechanisms, for example a second transcription factor, or feed‐forward loops, are needed to ensure the correct timing of expression. Such regulation could be via interactions with other factors, possibly other bHLHs, since bHLHs frequently function as dimers (Toledo‐Ortiz *et al*., 2003; Li *et al*., 2006). AMS interacts with bHLH89, bHLH91 and ATA20 proteins (Xu *et al*., 2010; Zhu *et al*., 2015); DYT1 interacts with itself, AMS, bHLH10, bHLH89, bHLH91, as well as four other bHLH which so far have no known function in flower development (Feng *et al*., 2012; Zhu *et al*., 2015; Cui *et al*., 2016). Cui *et al*. (2016) have shown that interaction of DYT1 with bHLH10/89/91 is required for its nuclear localization and function, and that the DYT1 bHLH89 heterodimer activates *TDF1*. Microarray analysis of *dyt1* compared to a triple bHLH mutant (*bhlh10/89/90)* showed that although there are shared targets the majority were not shared (707 downregulated in the triple bHLH mutant and 299 in *dyt1*) (Zhu *et al*., 2015). This indicates that the bHLH89/91/10 have distinct functions separate from DYT1 and this may be due to multiple binding partners of the bHLHs determining the regulation of downstream events. In our model, competitive complex formation of bHLHs with DYT1 and AMS to activate downstream targets (TDF1 and MYB80), improved the model fit, suggesting that other transcription factors, such as these bHLHs, may be driving downstream expression. Recently the *AMS* rice orthologue *TAPETUM DEGENERATION (TDR)* has been shown to contain six phosphorylation sites (Ye *et al*., 2015), which are important for protein–protein binding, and could be a way of regulating particular binding partners for modulation of downstream gene expression.

Although competitive binding with the bHLHs can create the observed dynamics, alternatives are possible. One option is that both DYT1 and AMS interact with other proteins to modulate expression (Xu *et al*., 2010; Feng *et al*., 2012). AMS may interact with downstream transcription factors to allow feed‐forward regulation (Fig. 7). In the re‐analysis of *ams* microarray data (Xu *et al*. (2010); Table S3), 69 transcription factors were two‐fold differently regulated, four of which are associated with pollen wall development (bHLH89, ANACO25, WUSCHEL (WUS), MYB99 (Xu *et al*., 2014)) and could be potential involved in feed‐forward loops. MYB99, for example, is upregulated 24 h and bHLH89 is upregulated 8 h after AMS DEX induction. Feed‐forward regulation is observed in the AMS rice orthologue TDR; the bHLH factor TIP2 directly regulates *TDR* and *ETERNAL TAPETUM* (*EAT1*) and interacts with TDR for tapetal development (Fu *et al*., 2014). TDR promotes expression of *EAT1* and also interacts with EAT1, to regulate tapetum PCD (Niu *et al*., 2013); however it is also possible is that DYT1‐AMS heterodimers may co‐regulate distinct genes from those regulated by DYT1 or AMS separately (Feng *et al*., 2012). DYT1 and AMS protein expression overlaps within the tapetum at anther stages 6, 7 (Gu *et al*., 2014), thus they may interact to activate early AMS targets such as MYB80 and DRL1 (Fig. 7). AMS has also been linked to chromatin modification via interaction with the SET‐domain protein ASH1‐RELATED3 (ASHR3) (Thorstensen *et al*., 2008). However, chromatin modification and ASHR3‐interaction alone cannot explain the dual role of AMS; this hypothesis is also not supported by the modelling, because poor data fit was seen using only transcriptional regulation.

This work presents the first report of biphasic AMS protein expression, in both early and late stages of pollen development, which define two distinct functional roles for AMS. We have shown that expression solely at either stage is insufficient for functional pollen development. Our mathematical network model explains how these two peaks can form based upon competitive interaction between AMS and other factors, potentially tapetum‐specific bHLH proteins, and presents a role for MS1 in the regulation of *AMS* RNA levels (possibly via chromatin remodelling), but also via an effect on AMS protein concentrations.

Our data demonstrate the early role of AMS in anther development, during tetrad progression to single microspore/polarized microspore stage. Analysis of known AMS targets shows that there is a delayed induction of the majority of the genes involved in pollen wall formation after DEX induction. The modelling reveals that our predicted network can create the biphasic peak seen in AMS‐GR‐YFP, and provides mechanistic insight into the key regulatory interactions required to control the network dynamics. This work indicates a complex network of feed‐back loops and regulation (Fig. 7) that are critical for controlling temporal gene expression in the anther tapetum for viable pollen production.

## Author contributions

Conceptualization Z.A.W.; methodology A.C.F., Z.A.W. and S.P.; software S.P.; investigation A.C.F., C.Y. and Z.Y.; resources I.F.; formal analysis S.P., L.R.B. and J.K. Funding acquisition Z.A.W. and D.Z.; visualization A.C.F. and S.P.; writing – original draft A.C.F., Z.A.W. and S.P.; writing – review and editing A.C.F., Z.A.W., S.P. and L.R.B.; supervision J.K., D.Z. and Z.A.W.

## Supporting information

Please note: Wiley Blackwell are not responsible for the content or functionality of any Supporting Information supplied by the authors. Any queries (other than missing material) should be directed to the *New Phytologist* Central Office.

**Fig. S1** DAPI staining of anthers to determine staging in PG228_PAMS:AMS‐GR‐YFP in Col background 24 h after DEX addition (Fig. 2).
**Fig. S2** Characterization of *amsms1* double mutant.
**Fig. S3** Expression of AMS‐YFP and MS1‐YFP.
**Fig. S4** qRT‐PCR expression of anther transcription factors in different male sterile mutant backgrounds.
**Fig. S5** Gene expression of AMS targets.
**Table S1** List of primers used
**Methods S1** Description of the mathematical model.

**Table S2** Expression analysis and protein intensity data of genes used for modelling

**Table S3** Re‐analysis of the microarray data of Xu *et al*. (2010)
