# Supplementary material for: Biphasic regulation of the transcription factor ABORTED MICROSPORES (AMS) is essential for tapetum and pollen development in Arabidopsis
Source: New Phytol. 2016 Oct 27;213(2):778–90. doi: 10.1111/nph.14200 (PMC5215365; doi:10.1111/nph.14200)
Supplement: Supplementary file 1 — Fig. S1 DAPI staining of anthers to determine staging in PG228_PAMS:AMS‐GR‐YFP in Col background 24 h after DEX addition (Fig. 2). Fig. S2 Characterization of amsms1 double mutant. Fig. S3 Expression of AMS‐YFP and MS1‐YFP. Fig. S4 qRT‐PCR expression of anther transcription factors in different male sterile mutant backgrounds. Fig. S5 Gene expression of AMS targets. Table S1 List of primers used Methods S1 Description of the mathematical model. [file NPH-213-778-s001.pdf]

Supporting Information Figures

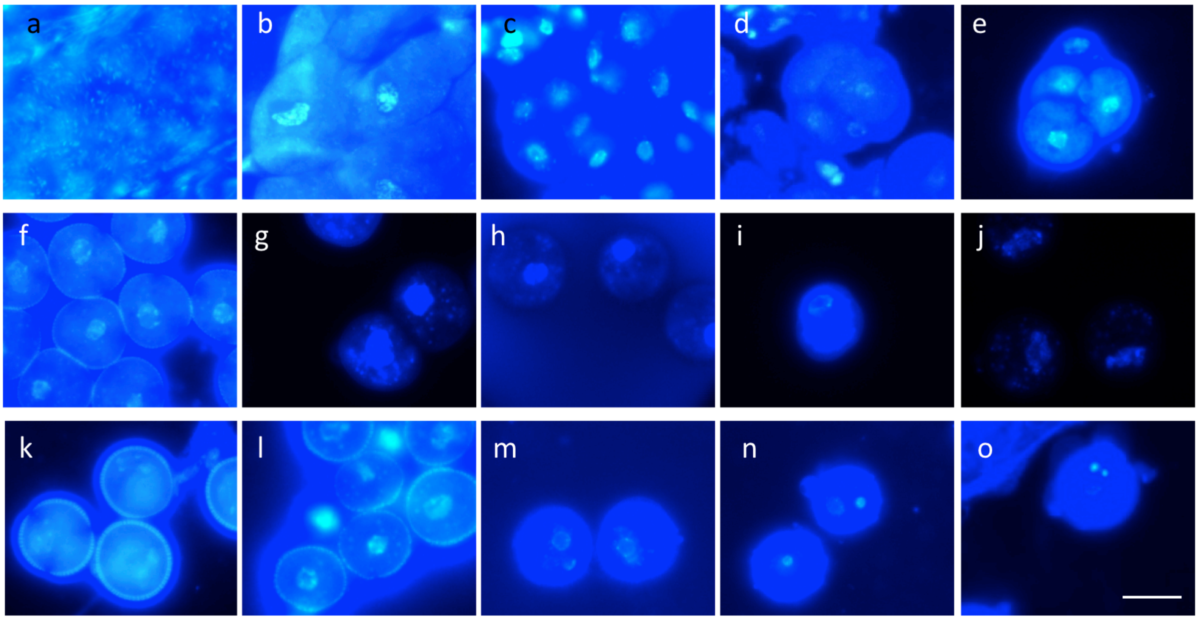

Figure S1: DAPI staining of *Arabidopsis* anthers to determine staging in PG228_PAMS:AMS-GR-YFP in Col background 24 hours after DEX addition (Fig 2). (a) Pollen Mother Cell (PMC) to Pre-Meiosis (Anther stage 5), (b) PMC – Meiosis (Anther stage 6), (c) PMC – Meiosis (Anther stage 6), (d-e) Tetrad (Anther stage 7), (f-h) Single Microspore (Anther stage 8), (i) Polarised Microspore (Anther stage 9), (j-l) Mitosis I (Anther stage 10), (m-n) Bicellular (Anther stage 11), (o) Tricellular (Anther stage 12). Representative image based on three replicates. Scale bar, 20 µm.

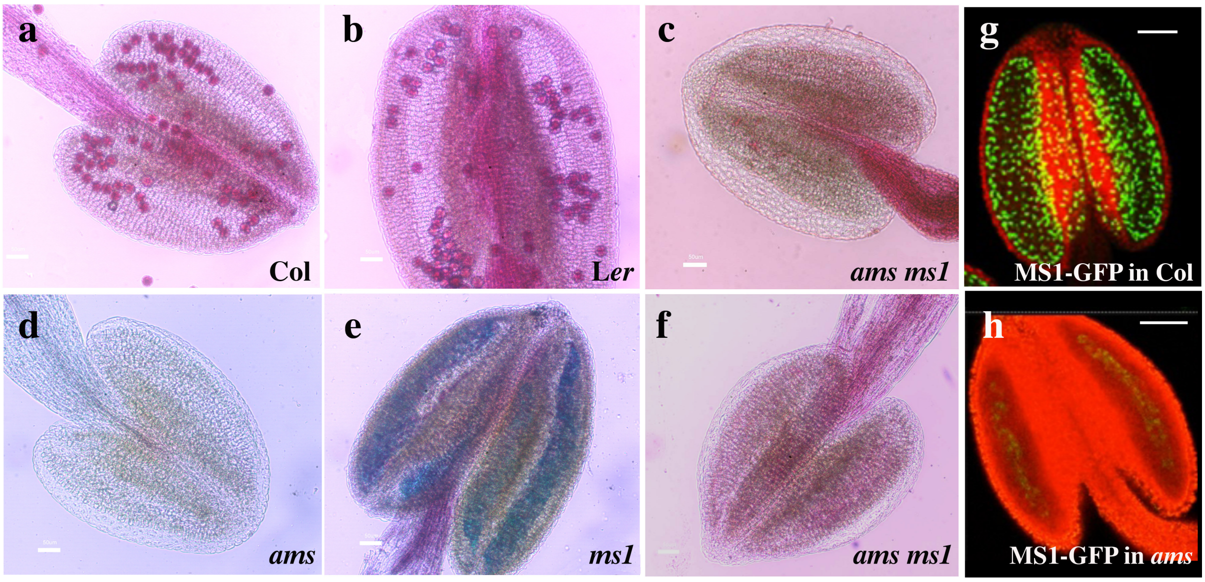

Figure S2: **Characterisation of *Arabidopsis thaliana amsms1* double mutant.** (a-f) Alexander staining showing pollen viability in (a) wild type Col, (b) wild type L*er*, (c) and (f) *ams ms1* double mutant, (d) *ams* (Col background), (e) *ms1* (L*er* background). The *ams ms1* double mutant exhibited the *ams* phenotype of early microspore degeneration. (g-h) Expression of pMS1::MS1-GFP in (g) wildtype (Col) and (h) *ams* mutant. Scale bar, 50 µm

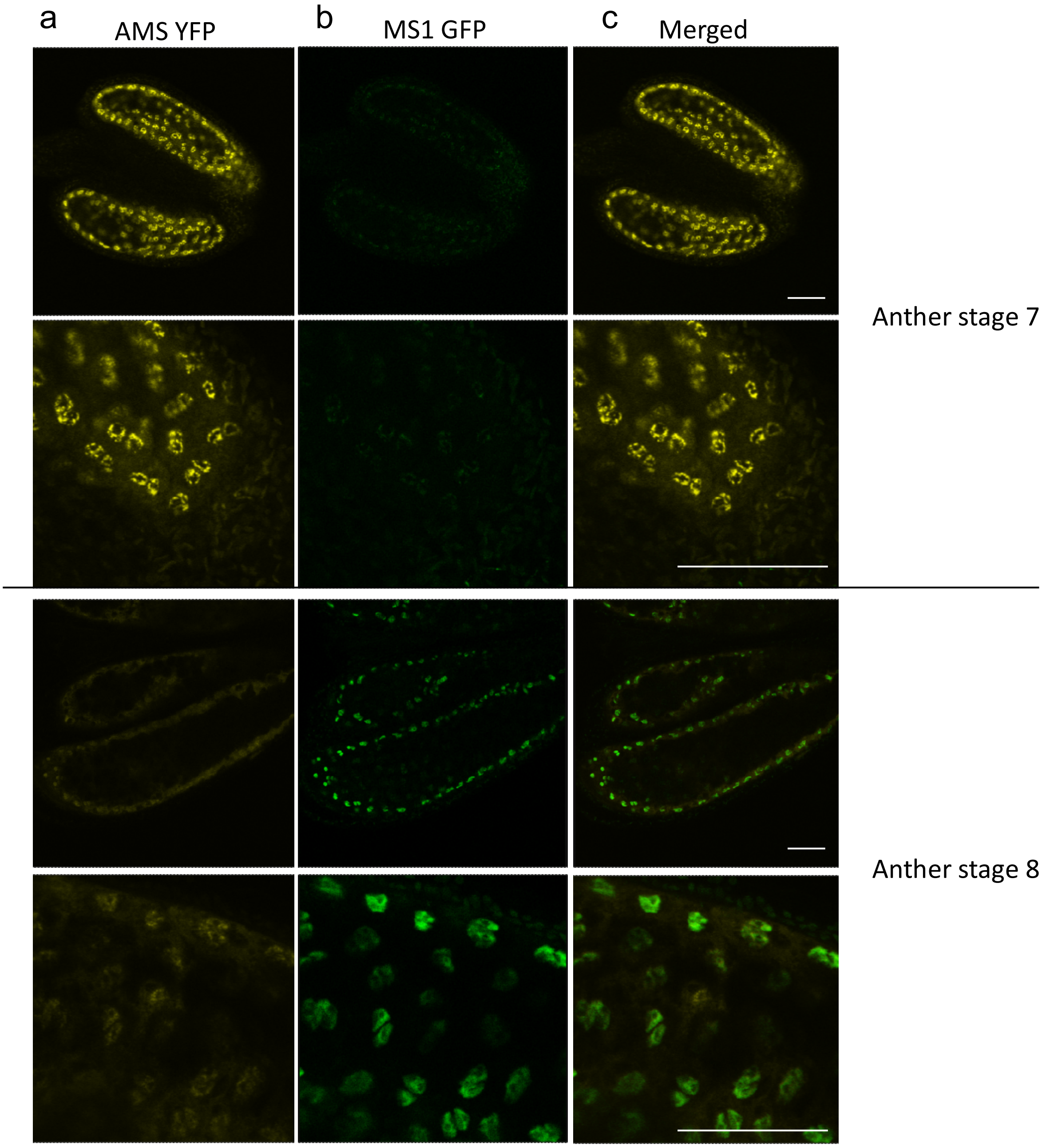

Figure S3: Expression of AMS-YFP and MS1-YFP in *Arabidopsis* anthers. (a) AMS-YFP, (b) MS1-GFP, and (c) merged image, in PG228_PAMS:AMS-GR-YFP crossed with PMS1:MS1-GFP in Col background, 24 hours after DEX treatment at Anther stage 7 and Anther stage 8. Representative image based on at least 8 anthers over 2 separate experiments. Scale bar, 10 µm. Extra images to support figure 3.

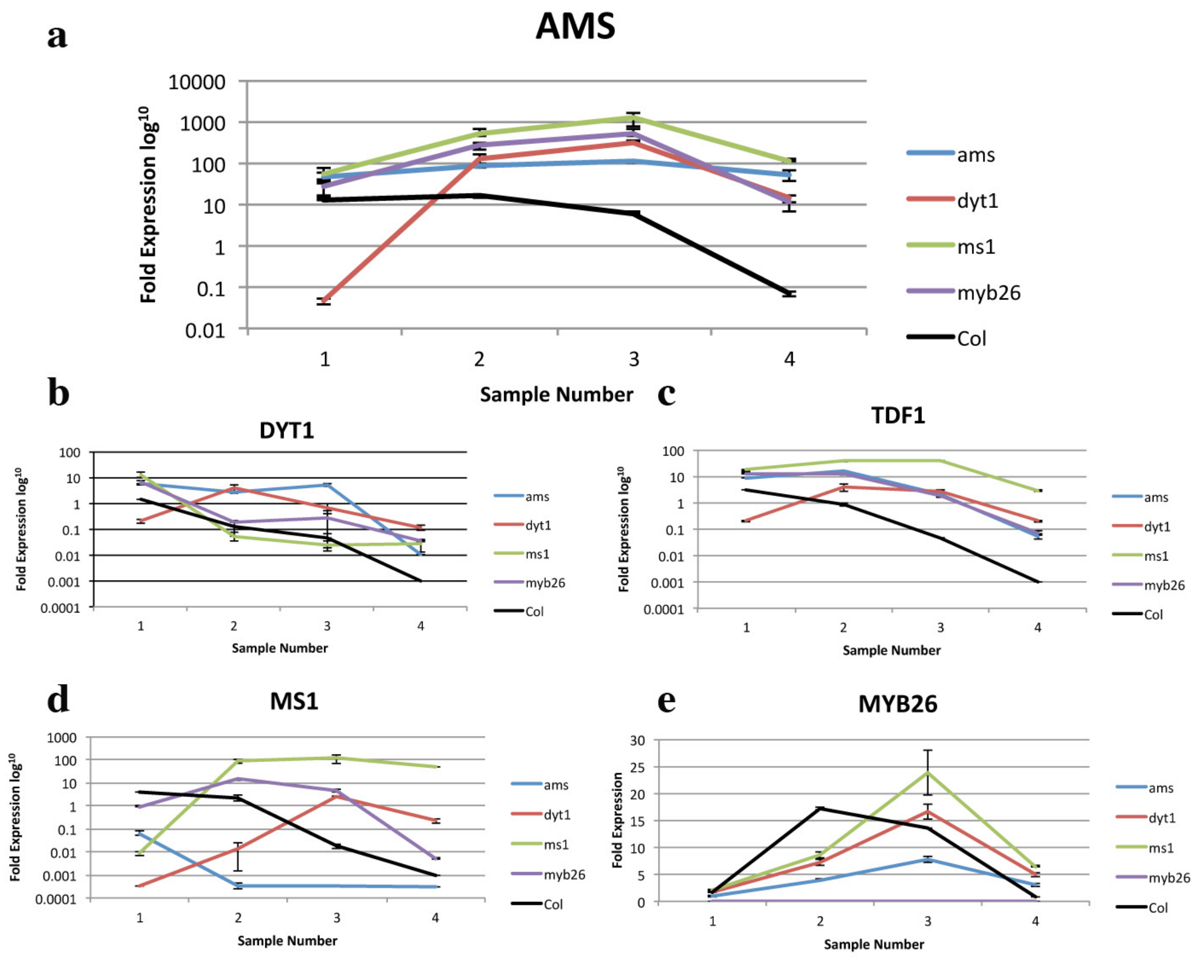

Figure S4: **qRT-PCR expression of anther transcription factors in different *Arabdiopsis* male sterile mutant backgrounds**. (a) *AMS*, (b) *DYT1,* (c) *TDF1*, (d) *MS1* and (e) *MYB26* in the different male sterile mutant backgrounds containing PG228_PAMS:AMS-GR-YFP construct. Sample 1; Anther stages 1-6, 2; Anther stage 7, 3; Anther stage 8-11, 4; Anther stage 12-13. Data are representative of means ±SE.

Gene	0hr	2hr	4hr	8hr	24hr	48hr	72hr	96hr	Direct Targets
TDF1									*ChIP*
TCP TF									
CEP1									
FUT6									
MYB80									*ChIP*
DRL1/TKPR1									ChIP
bHLH89									Y2H/IP
bHLH91									Y2H/IP
bHLH10									
ASHR3									Y2H
At5g07230									
LCR66									
MS2									
CYP722A1									
LTP - At5g07230									
LAP5									ChIP
CYP703A2									ChIP
CYP704B1									ChIP
MS1									
MYB99									
WBC27									ChIP
KCS7									ChIP
GRP18									ChIP
LTP12									ChIP
QRT3									ChIP
ATA20									Y2H/IP
At1g28375									
PHT6									
CYP86C2									
At1g59740									ChIP
At3g15900									
GRP14									
GRP19									ChIP
GDSL									
XTH3									
ANAC025									
ATA27									
EXL6									ChIP+EMSA
GRP20									
At3g07820									
AGP40									
At5g19580									
At1g04770									

Figure S5: **Gene expression of AMS targets.** Expression of AMS targets over a DEX inducible time-course in PG228_PAMS:AMS-GR-YFP in *ams* background treated daily with DEX. Each gene expression is shown individually normalised to expression at 0hr in PG228_PAMS:AMS-GR-YFP in *Col* background, and highest expression for that gene is shown as a full bar, the other values are a percentage representative of the highest value. Direct AMS targets are labelled by the experiment (ChIP/EMSA). Black coloured bars represent those genes that are reduced after DEX treatment and grey coloured bars represent those genes that are up-regulated after DEX treatment. Data are representative of means ±SE.

Supporting Tables

Table S1: List of primers used.

Name	Reason	Sequence (5’..3’)
AMSprom-Kpn1_F1	Cloning of AMS promoter and AMS gene	cccGGTACCGTATGGATATGTGTATTCATGGATAG
AMS_cDNA_AVRII_R1	Cloning of AMS promoter and AMS gene	ggcctaggTTGGTTGTGGTAATGGTTGATGTGTTG
AMS_a_F	Transgenic Plant genotyping	GCAGAATGAGGCAAAGGAG
GFP_PGWB5_R	Transgenic Plant genotyping	AAGTCGTGCTGCTTCATGTG
PG0229 F2	Transgenic Plant genotyping	ATTATGCCACACAAGGAGCAC
AMS PRO R4	Transgenic Plant genotyping	CTCAGCTCAAATCGCAGCTTGTGTCATTAAC
DBAMS_1F	Ams SALK insert genotyping	CACCatggagagtaatatgcaaaacttgttgg
DBAMS_7R	AMS SALK insert genotyping	TCCTGACTTCTTCTTGTACTTTGGATC
LBb1.3R	AMS SALK insert genotyping	ATTTTGCCGATTTCGGAAC
DBAMS4F	AMS qRT-PCR	CACCaaaggatctcaagccaagaacctg
DBAMS3R	AMS qRT-PCR	GTTCTCTTCAAGCTCGTCTTGAAGCTCC
RRT	MS1 qRT-PCR	CAGCCTCAACTCCATTCCTT
LMRNAsp	MS1 qRT-PCR	CCATTGCCAATATGTTGGTTG
4g21330_DYT_1F	DYT1 qRT-PCR	ATGGGTGGAGGAAGCAGATTTC
4g21330_DYT_406R	DYT1 qRT-PCR	CCCCAATCTTACACAATTGCACA
TDF1_F	TDF1 qRT-PCR	CGGACCCGGTTCCTCAAGTA
TDF1_R	TDF1 qRT-PCR	ACGAAGGAACTCGCGGAATG
At3g13220_624F	WBC27 qRT-PCR	CAAAGAGCTCGGCCTCGAAA
At3g13220_934R	WBC27 qRT-PCR	TGCCGTAAAAGGCAGGGTGT
CYP703A2 885F	CYP703A2 qRT-PCR	TGAGAACGGGAAGGCACACA
CYP703A2 1188R	CYP703A2 qRT-PCR	ACGTGTCTTTGCCGGGATGT
PP2A3 1255F	PP2A qRT-PCR	TCCGTGAAGCTGCTGCAAAC

PP2A3 1677R	PP2A qRT-PCR	CACCAAGCATGGCCGTATCA
LTP12_53F	LTP12 qRT-PCR	TGGCATCCCCAACAGAGTCA
LTP12_315R	LTP12 qRT-PCR	GGGGTAGGGAATTGAAACACCA
MYB103_437F	MYB103 qRT-PCR	TCGAGGCCTCTACCACCACAA
MYB103_177R	MYB103 qRT-PCR	TGAAACGGCTCTTGCTGACG
EXL6_562F	EXL6 qRT-PCR	TGTCGCTCCCGCTCTTCTTC
EXL6_742R	EXL6 qRT-PCR	TCCGAATACCCCACCGAATG
QRT3_F	QRT3 qRT-PCR	5'-CGGACAAGCATAGACAAC-3'
QRT3_R	QRT3 qRT-PCR	5'-CGGTTATGTGTTGACCAA-3'
KCS7_339F	KCS7 qRT-PCR	GTGCCTGCGACAGTCCATGA
KCS7_587R	KCS7 qRT-PCR	GCAGCCCATACCGCTCAAAC
A6_F	A6 qRT-PCR	5'-TCCACCGTGTATTCAATC-3'
A6_R	A6 qRT-PCR	5'-GATCCTCACAATGCCTTA-3'
GRP18RTF	GRP18 qRT-PCR	TTCCACCCCCTGGTTTATTT
At5g07520R	GRP18 qRT-PCR	GATGGCCGTTGCTCTCAT
bHLH10_957F	bHLH10 qRT-PCR	TGGTGAAGGAGGAGGAGGAGAA
bHLH10_1246R	bHLH10 qRT-PCR	CACCAATCTGTCCACCTGCAAC
1g06170_929F	bHLH89 qRT-PCR	5'-CCTTGAGATGCTCATGGCTCA-3'
1g06170_1118R	bHLH89 qRT-PCR	5'-TGGTGCTCTCCGATTTGTGC-3'
2g31210_927F	bHLH91 qRT-PCR	5'-GAAGAAGCCGGAGAGCGATG-3'
2g31210_1144R	bHLH91 qRT-PCR	5'-CTCCGGCAACATGGTGAAGA-3'
R1451	MYB26 qRT-PCR	TTGGGAACATCAATGGGGTCAT
RT345	MYB26 qRT-PCR	GCTCATGTCTCACCATCATCACG
NST1_66F	NST1 qRT-PCR	TCATCCGACCGAGGAAGAGC
NST1_1392R	NST1 qRT-PCR	GAAGCTCCTCCGACGGGACT
NST2_F	NST2 qRT-PCR	CCAAGTCAACAACTGCCACG
NST2_R	NST2 qRT-PCR	GTCGTTCTCGCGAAATCTGC
MYB99_122F	MYB99 qRT-PCR	GAGGATGGTGCTGGAGAGAC
MYB99_444R	MYB99 qRT-PCR	GCTTTGGATCGTTGACCAAT
YFP_NY-F	YFP qRT-PCR	AGCAAGGGCGAGGAGCTGTTC
GFP_PGWB5_R	YFP qRT-PCR	AAGTCGTGCTGCTTCATGTG

LAP6_390F	LAP6 qRT-PCR	ATTGCAAACGAGGCGGTTGT
LAP6_617R	LAP6 qRT-PCR	ACGGAGGCCAGTCACACCTC
ATA20_682_F	ATA20 qRT-PCR	TGGTGTTGGCTATGGCGTTG
ATA20_943_R	ATA20 qRT-PCR	ACCTGGAGCTCCCGTGACAG
ATBCB_205_F	AtBCB qRT-PCR	CTGCTGGGAGGCATGATGTG
ATBCB_459_R	AtBCB qRT-PCR	TTGGGGTTGATCCAGGTGCT
Lip3_485F	At5g24210 qRT-PCR	TGCGTTACGGGGAACTGTCC
Lip3_668R	At5g24210 qRT-PCR	TGCCAGGCCAGCTCCTAAAG
1g28375_88_F	At1g28375 qRT-PCR	TCGACCGTAATGGCAAGACC
1g28375_257_R	At1g28375 qRT-PCR	ACTGGCCCGGGATAAGGAAA
CYP81_718F	CYP81F2 qRT-PCR	GTGCGAGCCATCCAGGAGAT
CYP81_996R	CYP81F2 qRT-PCR	CCATCGCCCATTCCAATGTT
PHT6_231F	PHT6 qRT-PCR	GGCCTTCGCTGGAACGTTTA
PHT6_529R	PHT6 qRT-PCR	TCCCAATCCCTTGCATACCG
CYP86C2_709F	CYP86C2 qRT-PCR	CCGCCTTTTGTGTGGAAACC
CYP86C2_1002R	CYP86C2 qRT-PCR	AGTAAGCGCGACGGAACTCG
1g59740_511F	At1g59740 qRT-PCR	TTCGGCTTCGTCGAGCTTTC
1g59740_800R	At1g59740 qRT-PCR	TCGCCCATTGAGAATGCAAA
LCR66_137F	LCR66 qRT-PCR	TGGGTCTGGTCACGGTAGAGG
LCR66_291R	LCR66 qRT-PCR	GGTGCACAAACAGCGACGAC
3g07820_196F	At3g07820 qRT-PCR	ACGGAGGAGGAGCCTTCGAC
3g07820_621R	At3g07820 qRT-PCR	CTGTCTTCTGGGGCGACGAT
3g15900_244F	At3g15900 qRT-PCR	GGAGTTCCCACGCTTTCTCG
3g15900_413R	At3g15900 qRT-PCR	GCTTGTTCCGACCGGTCATC
CYP89A6_1073F	CYP89A6 qRT-PCR	ACGCGCAAAAGATGCCGTAT
CYP89A6_1268R	CYP89A6 qRT-PCR	GCCATTGGTTCCTCCCACAC
CBF1_378F	CBF1 qRT-PCR	TTTGGCTCGGGACTTTCCAA
CBF1_659R	CBF1 qRT-PCR	ACGCACCTTCGCTCTGTTCC
XTH3_90F	XTH3 qRT-PCR	TTTCGGAGGACGAGGAATCG
XTH3_247R	XTH3 qRT-PCR	CGTAGGCGTCCTTGGATTCG
LTP_119F	At5g07230 qRT-PCR	CCCAGCAGTGCAGAGACGAA

LTP_274R	At5g07230 qRT-PCR	GTGTGGTGGCTGCTCGAAGA
3g20865_92F	AGP40 qRT-PCR	TCTTTGTGGCCCTCTTTATCTCG
3g20865_248R	AGP40 qRT-PCR	GCCAAGAAGGCGGAGAGTGA
ATA27_731F	ATA27 qRT-PCR	TGTGTCACGATGGACGGTCA
ATA27_1016R	ATA27 qRT-PCR	GGCAGTCGATGTCCGATGTG
5g19580_202F	At5g19580 qRT-PCR	CCTCCCTGGGTTCAACAACG
5g19580_387R	At5g19580 qRT-PCR	GCATGCATCCCTGACACACC
TDF1_ChIP_F1	TDF1 ChIP + EMSA	TGTGGTTAGACAAAGTAGCAGC
TDF1_ChIP_R1	TDF1 ChIP + EMSA	TCACCATTCTCTGATCCGCA
DYT1_ChIP_F1	DYT1 ChIP + EMSA	GAACCAATCTTTGCGGAGGG
DYT1_ChIP_R1	DYT1 ChIP + EMSA	GGAGACGAGCATTTTGTGGG
MYB80_ChIP_F2	MYB80 ChIP	CGGCTTAAGGGAATCACACG
MYB80_ChIP_R2	MYB80 ChIP	CAAAGATTGCCCCACCCAAA

Supporting Files

Method S1: Description of the mathematical model.

Table S2: Expression analysis and protein intensity data of genes used for modelling; original construction of the mathematical model and the data used to test the model.

Table S3: Re-analysis of the microarray data of Xu et al. (2010), showing genes which were differentially regulated at each stage.

Xu, J., Yang, C., Yuan, Z., Zhang, D., Gondwe, M.Y., Ding, Z., Liang, W., Zhang, D., and Wilson, Z.A. (2010). The ABORTED MICROSPORES Regulatory Network Is Required for Postmeiotic Male Reproductive Development in Arabidopsis thaliana. Plant Cell **22,** 91-107.

Biphasic regulation of the transcription factor ABORTED MICROSPORES (AMS) is essential for tapetal and pollen development in Arabidopsis.

Methods S1: Description of the Mathematical Model

In this supplementary text, we describe the development and analysis of a set of mathematical models of the AMS genetic regulatory network in the tapetum. The model describes the dynamics of the five network components (DYT1, TDF1, AMS, MYB80, MS1), which are involved in regulating many of the genes in the anther, including those responsible for pollen development and release. The model investigates the dynamics of these components during nine anther samples, corresponding to anther stages 5-12, and determines how the network dynamics create the two peaks of AMS that control tapetal development.

1 Model I

1.1 Model description

The initial model describes the transcription factor cascade of DYT1, TDF1, AMS, MYB80 and MS1 in that order, and consider both the mRNA and protein levels. The RT-qPCR data shows a peak of DYT1 expression at samples 2-3, suggesting that DYT1 transcription is regulated by upstream factors such as SPL/NZZ or EXS/EMS1; modelling these upstream components is beyond the scope of the current model, and therefore we prescribe the DYT1 transcription rate to be given by an exponential function of time:

$$\alpha_{mD}(t) = a_{mD} \exp^{-\left(\frac{t-b_D}{c_D}\right)^2},$$

where a_{mD}, b_D, c_D, are unknown parameters. As well as the direct cascade, we assume that MS1 negatively inhibits TDF1, AMS and MS1 transcription, and assume that AMS negatively inhibits TDF1 transcription, as described in the main text.

Additionally, we make the assumption that MS1 protein increases the rate at which AMS protein is degraded, which we take to be a direct interaction even though it is likely to be through an intermediate gene. An assumption such as this is required in order to decouple the protein and transcript behaviours for AMS, as is seen in the experimental data. These interactions may be seen in the network diagram given in Figure 4 of the main text. We take a deterministic approach, with the network dynamics describes by a coupled system of ordinary differential equations (ODEs). Using Hill functions to represent the transcriptional regulations, the complete system is given by

$$([\text{mDYT1}])' = \alpha_{mD}(t) - \mu_{mD}[\text{mDYT1}],$$

$$([\text{mTDF1}])' = \alpha_{mT}\frac{[\text{pDYT1}]}{K_T + [\text{pDYT1}] + [\text{pMS1}]\zeta_{TM} + [\text{pAMS}]\zeta_{TA}} - \mu_{mT}[\text{mTDF1}],$$

$$
\begin{aligned}
([\text{mAMS}])' &= \alpha_{mA}\frac{[\text{pTDF1}]}{K_A + [\text{pTDF1}] + [\text{pMS1}]\zeta_{AM}} - \mu_A[\text{mAMS}],\\
([\text{mMYB80}])' &= \alpha_{mY}\frac{[\text{pAMS}]}{K_Y + [\text{pAMS}]} - \mu_Y[\text{mMYB80}],\\
([\text{mMS1}])' &= \alpha_{mM}\frac{[\text{pMYB80}]}{K_M + [\text{pMYB80}] + [\text{pMS1}]\zeta_{MM}} - \mu_M[\text{mMS1}],\\
([\text{pDYT1}])' &= \alpha_{pD}[\text{mDYT1}] - \mu_{pD}[\text{pDYT1}],\\
([\text{pTDF1}])' &= \alpha_{pT}[\text{mTDF1}] - \mu_{pT}[\text{pTDF1}],\\
([\text{pAMS}])' &= \alpha_{pA}[\text{mAMS}] - \mu_{pA}[\text{pAMS}]\left(1 + \frac{\zeta_{pA}[\text{pMS1}]}{K_{pA} + [\text{pMS1}]}\right),\\
([\text{pMYB80}])' &= \alpha_{pY}[\text{mMYB80}] - \mu_{pY}[\text{pMYB80}],\\
([\text{pMS1}])' &= \alpha_{pM}[\text{mMS1}] - \mu_{pM}[\text{pMS1}],
\end{aligned}
$$

where α_{mi} are the maximum transcription rates, α_{pi} are the translation rates, and μ_{mi} and μ_{pi} are the mRNA and protein degradation rates respectively, for species i. In the Hill functions, parameters K_i are the half-saturation constants and ζ_{ij} is a parameter expressing the negative regulation of species i by species j. Throughout a subscript Y represents MYB80, otherwise the first letter of the gene name is used.

1.2 Parameter estimation

The model dynamics depend on 32 parameter values which are not available in the current literature; we therefore fit these parameter values using the experimental data presented in the main text. The predicted mRNA levels were fitted to a RT-qPCR DEX-inducible time-course of buds, using staged material from *Col*-0 (see Table S2). The data give the mRNA levels at 9 samples during anther development; these samples are assumed to be approximately evenly spaced in time and we therefore use them as a proxy for time in the governing equations. Additionally, the AMS and MS1 protein levels were fitted to the mean values of the protein levels obtained from confocal images of DEX-inducible AMSpro::AMS-GR-YFP x MS1pro::MS1-GFP lines and measured using the IMARIS software, averaged across tapetal cells (see Figure 2 of the main text, and Table S2), at equivalent timepoints as the RT-qPCR data. The other protein levels were unavailable and hence not included in the fits. In order to fit the parameters to these two sets of data, we use the MATLAB genetic algorithm (ga) to find the parameter set which minimizes the weighted squared distance between the model predictions and experimental data; thus, we set the algorithm to minimise the following objective function,

$$
f = \sum_{t=1}^{9}\left[\frac{2\left([\text{pAMS}](t) - \overline{[\text{pAMS}]}(t)\right)^2}{\max_t(\overline{[\text{pAMS}]})} + \frac{2\left([\text{pMS1}](t) - \overline{[\text{pMS1}]}(t)\right)^2}{\max_t(\overline{[\text{pMS1}]})} + \sum_X \frac{(\text{mX}(t) - \overline{\text{mX}}(t))^2}{\max_t(\overline{\text{mX}})}\right], \tag{1}
$$

where X is the set of transcription factors under consideration (DYT1, TDF1, AMS, MYB80, MS1, and in later models bHLH89/91) and barred quantities are the experimental data. Given our focus on the AMS protein dynamics, the objective function includes a two-fold weighting on the protein data compared to the mRNA data. All the parameters in the model were varied on a logarithmic scale and taken to be in the range 10^{-6} and 10^6. Preliminary fits were obtained using objective functions with (i) no weighting, (ii) weighting using the variance of each data measurement, and (iii) weighting using the maximum value in data set; we obtained the best fits using the maximum (option iii), as described in equation 1). The best fit found by 30 runs of the genetic algorithm had an objective function value of $f^* = 1.6734$, with the parameter values given in Table I.

The resulting plots are shown in Figure I; the model predictions and data are not in good agreement and the model does not reproduce the second peak in the AMS protein data.

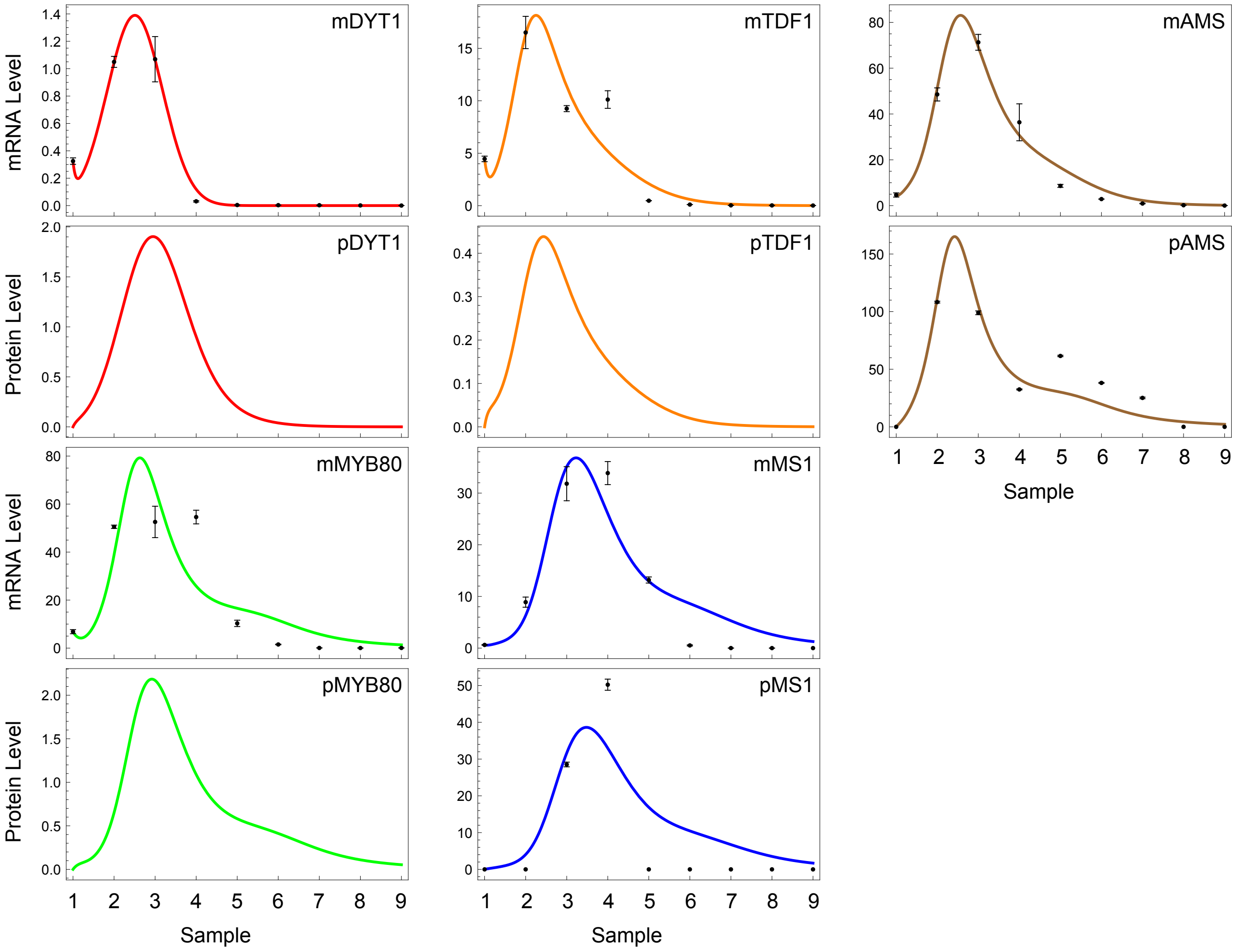

Figure I: Predicted (lines) and measured (dots with standard error bars) protein and mRNA levels for Model I, using the model parameter values given in Table I.

2 Model II

In order to improve the fit to the data, we added an additional transcription factor which works with DYT1 and AMS to regulate their targets; this is hypothesised to be another bHLH transcription factor such as bHLH89 or bHLH91, as they are known to bind to DYT1 (Feng et al., 2012) and AMS (Xu et al., 2010). In the same way as for DYT1, we assume this additional bHLH(s) is also regulated by some unknown upstream genes and so assume the rate of bHLH transcription to be given by

$$\alpha_{mB}(t) = a_{mB} \exp^{-\left(\frac{t-b_B}{c_B}\right)^2}, \tag{2}$$

introducing three further unknown parameters a_{mB}, b_B, c_B. The set of differential equations for the system is therefore given by:

$$\begin{aligned}
([\text{mDYT1}])' &= \alpha_{mD}(t) - \mu_{mD}[\text{mDYT1}],\\
([\text{mbHLH}])' &= \alpha_{mB}(t) - \mu_{mB}[\text{mbHLH}],\\
([\text{mTDF1}])' &= \alpha_{mT}\frac{[\text{pDYT1}][\text{pbHLH}]}{K_T + [\text{pDYT1}]\,[\text{pbHLH}] + [\text{pMS1}]\zeta_{TM} + [\text{pAMS}]\zeta_{TA}} - \mu_{mT}[\text{mTDF1}],\\
([\text{mAMS}])' &= \alpha_{mA}\frac{[\text{pTDF1}]}{K_A + [\text{pTDF1}] + [\text{pMS1}]\zeta_{AM}} - \mu_A[\text{mAMS}],\\
([\text{mMYB80}])' &= \alpha_{mY}\frac{[\text{pAMS}][\text{pbHLH}]}{K_Y + [\text{pAMS}][\text{pbHLH}]} - \mu_Y[\text{mMYB80}],\\
([\text{mMS1}])' &= \alpha_{mM}\frac{[\text{pMYB80}]}{K_M + [\text{pMYB80}] + [\text{pMS1}]\zeta_{MM}} - \mu_M[\text{mMS1}],\\
([\text{pDYT1}])' &= \alpha_{pD}[\text{mDYT1}] - \mu_{pD}[\text{pDYT1}],\\
([\text{pbHLH}])' &= \alpha_{pB}[\text{mbHLH}] - \mu_{pB}[\text{pbHLH}],\\
([\text{pTDF1}])' &= \alpha_{pT}[\text{mTDF1}] - \mu_{pT}[\text{pTDF1}],\\
([\text{pAMS}])' &= \alpha_{pA}[\text{mAMS}] - \mu_{pA}[\text{pAMS}]\left(1 + \frac{\zeta_{pA}[\text{pMS1}]}{K_{pA} + [\text{pMS1}]}\right),\\
([\text{pMYB80}])' &= \alpha_{pY}[\text{mMYB80}] - \mu_{pY}[\text{pMYB80}],\\
([\text{pMS1}])' &= \alpha_{pM}[\text{mMS1}] - \mu_{pM}[\text{pMS1}].
\end{aligned}$$

This model is fitted to the mRNA and protein levels as described for the previous Model I. As bHLH89 and bHLH91 have very similar expression patterns and protein structure and have previously been shown to act redundantly, we assumed they may be acting redundantly and so the transcript levels of the additional bHLH were also fit to the sum of the measured levels of bHLH89 and bHLH91 mRNA; this the difference between predicted and measured bHLH mRNA levels was included as an additional term in the objective function. We note that other transcription factors, such as bHLH10, could also contribute to the modelled bHLH levels, though have not been included here. The best fit has an objective function value of $f^* = 1.3869$, with the parameter values given in Table I and model dynamics shown in Figure II. This model does include the second peak in the AMS protein, and is a much better fit than Model II.

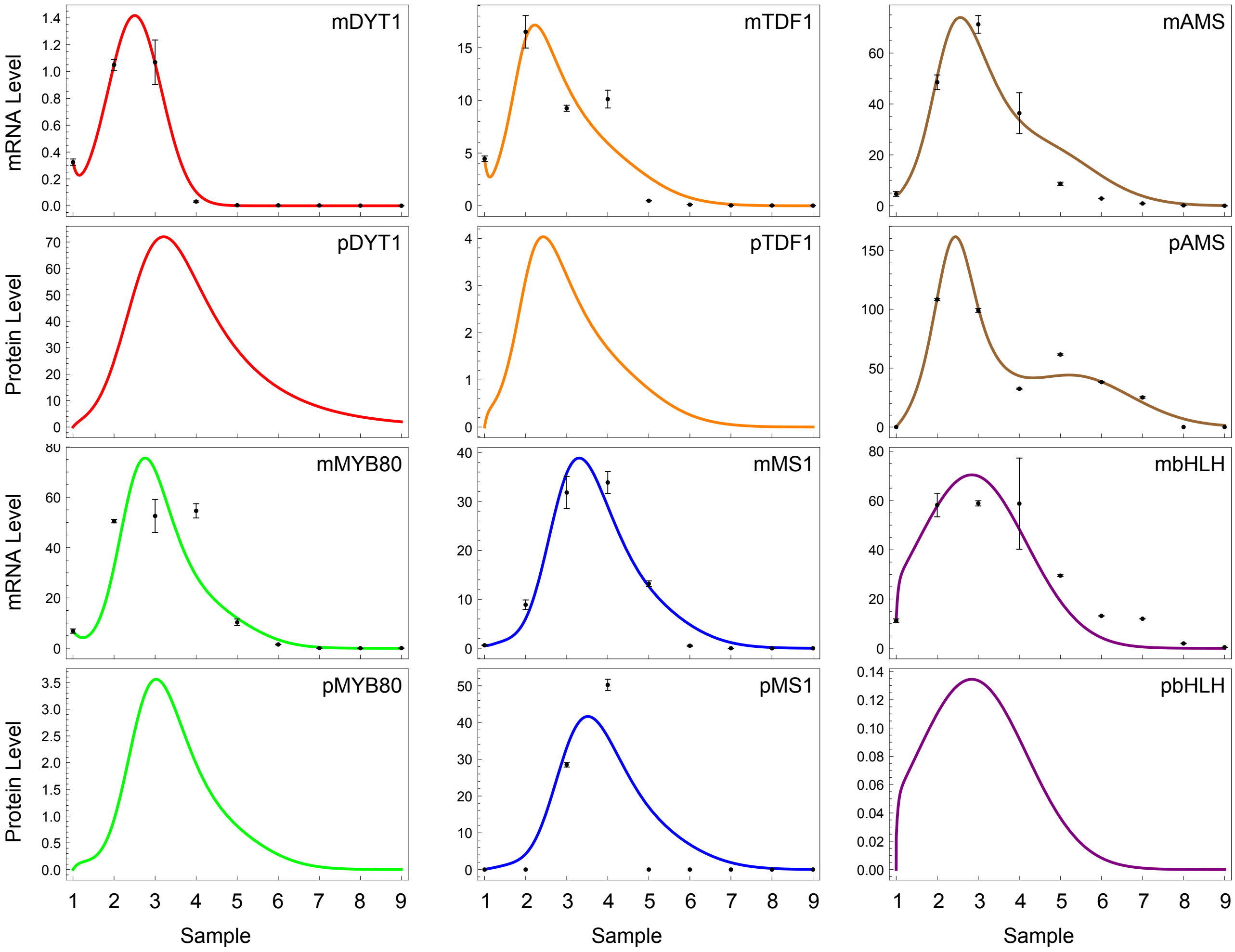

Figure II: Predicted (lines) and measured (dots with standard error bars) protein and mRNA levels for Model II, using the model parameter values given in Table I.

3 Model III

To further improve the model fit, we now assume that the additional bHLH forms protein complexes with DYT1 and AMS to promote the regulation of TDF1 and MYB80 respectively. The final set of equations is therefore given by,

$$\begin{aligned}
([\text{mDYT1}])' &= \alpha_{mD}(t) - \mu_{mD}[\text{mDYT1}],\\
([\text{mbHLH}])' &= \alpha_{mB}(t) - \mu_{mB}[\text{mbHLH}],\\
([\text{mTDF1}])' &= \alpha_{mT}\frac{[\text{DYT1:bHLH}]}{K_T + [\text{DYT1:bHLH}] + [\text{pMS1}]\zeta_{TM} + [\text{pAMS}]\zeta_{TA}} - \mu_{mT}[\text{mTDF1}],\\
([\text{mAMS}])' &= \alpha_{mA}\frac{[\text{pTDF1}]}{K_A + [\text{pTDF1}] + [\text{pMS1}]\zeta_{AM}} - \mu_A[\text{mAMS}],\\
([\text{mMYB80}])' &= \alpha_{mY}\frac{[\text{AMS:bHLH}]}{K_Y + [\text{AMS:bHLH}]} - \mu_Y[\text{mMYB80}],\\
([\text{mMS1}])' &= \alpha_{mM}\frac{[\text{pMYB80}]}{K_M + [\text{pMYB80}] + [\text{pMS1}]\zeta_{MM}} - \mu_M[\text{mMS1}],\\
([\text{pDYT1}])' &= \alpha_{pD}[\text{mDYT1}] - \mu_{pD}[\text{pDYT1}] - \alpha_{DB}[\text{pDYT1}][\text{pbHLH}] + \mu_{DB}[\text{DYT1:bHLH}],\\
([\text{pbHLH}])' &= \alpha_{pB}[\text{mbHLH}] - \mu_{pB}[\text{pbHLH}] - \alpha_{AB}[\text{pAMS}][\text{pbHLH}] + \mu_{AB}[\text{AMS:bHLH}]\\
&\quad - \alpha_{DB}[\text{pDYT1}][\text{pbHLH}] + \mu_{DB}[\text{DYT1:bHLH}],\\
([\text{pTDF1}])' &= \alpha_{pT}[\text{mTDF1}] - \mu_{pT}[\text{pTDF1}],\\
([\text{pAMS}])' &= \alpha_{pA}[\text{mAMS}] - \mu_{pA}[\text{pAMS}]\left(1 + \frac{\zeta_{pA}[\text{pMS1}]}{K_{pA} + [\text{pMS1}]}\right)\\
&\quad - \alpha_{AB}[\text{pbHLH}][\text{pAMS}] + \mu_{AB}[\text{AMS:bHLH}],\\
([\text{pMYB80}])' &= \alpha_{pY}[\text{mMYB80}] - \mu_{pY}[\text{pMYB80}],\\
([\text{pMS1}])' &= \alpha_{pM}[\text{mMS1}] - \mu_{pM}[\text{pMS1}],\\
([\text{AMS:bHLH}])' &= \alpha_{AB}[\text{pAMS}][\text{pbHLH}] - \mu_{AB}[\text{AMS:bHLH}],\\
([\text{DYT1:bHLH}])' &= \alpha_{DB}[\text{pDYT1}][\text{pbHLH}] - \mu_{DB}[\text{DYT1:bHLH}].
\end{aligned}$$

This model fits well to the experimental data, including the second peak of the AMS protein, with the best fit having an objective function value of $f = 0.8129$. The parameters are given in Table I and the model dynamics are shown in Figure III, showing how the predicted AMS protein now exhibits two peaks.

Looking at the best fit from this model, the DYT1-bHLH complex associates faster than the AMS-bHLH complex, delaying the main production of MYB80 and MS1 until after the DYT1 levels begin to drop. Once MS1 levels rise, the levels of AMS protein are quickly down-regulated. This decreased level of AMS protein reduces MS1 protein, causing a short-lived spike in AMS protein levels before the negative feedback of TDF1 along with the lack of DYT1 causes all elements in the model to decay to zero at the end of the timecourse.

As shown in II, the Akaike Information Criterion with correction for finite sample size (AICc), shows that Model III is a better fit than the other two models, even though it includes more parameters.

To evaluate the robustness of this fit, we used the HYPERSPACE algorithm (Zamora-Sillero et al., 2011) to assess the global parameter sensitivity. This algorithm uses an out-of-equilibrium adaptive Metropolis Monte Carlo method to identify regions of parameter space that satisfy a prescribed criteria. Here, we set the algorithm to search for parameter combinations which correspond to predicted dynamics with an objective function value within 10% of the best value, i.e. with $f^* < 0.8942$. In this case, the algorithm produces 17,742

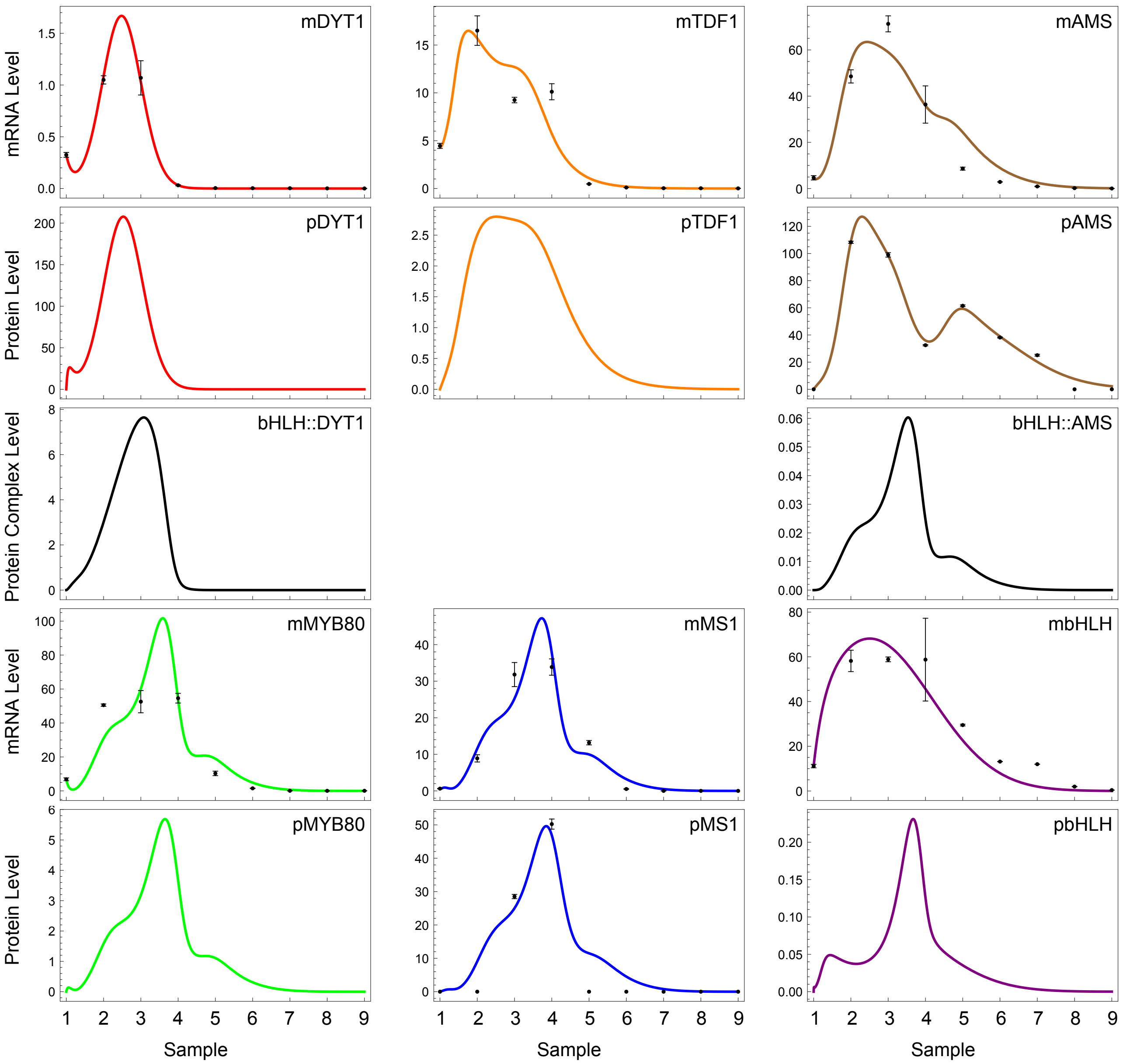

Figure III: Predicted (lines) and measured (dots with standard error bars) protein and mRNA levels for Model III, using the model parameter values given in Table I.

parameter sets, and Figure IV shows boxplots of the parameter ranges within these sets. The majority of the parameter values can be considered to be fairly well constrained by the experimental data, with the exception of three negative feedback parameters, $\zeta_{TA}, \zeta_{MM}, \zeta_{AM}$. These three parameters vary over a large range but are small in all the parameter sets, suggesting that these negative feedback loops (AMS inhibiting TDF1 transcription, MS1 inhibiting AMS transcription and MS1 inhibiting its own transcription) are not required for the model.

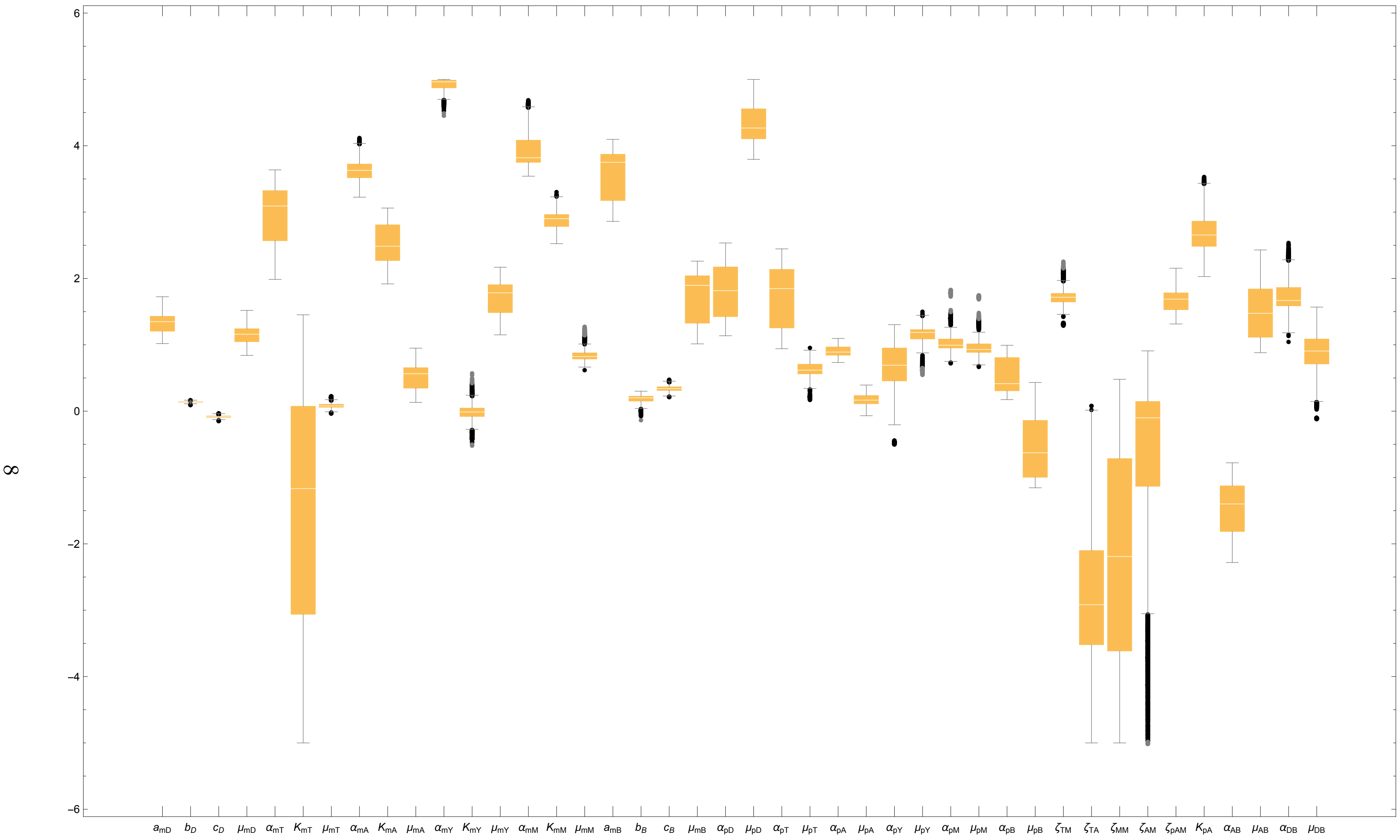

Figure IV: Set of boxplots from running the HYPERSPACE algorithm, showing the variability of the 42 parameters in Model III, for parameter combinations that achieve a similarly good fit as the set of values in Table I.

4 Model IV

Our fourth model is therefore one given by removing these three negative feedback loops (AMS inhibiting TDF1 transcription, MS1 inhibiting AMS transcription and MS1 inhibiting its own transcription), this does not significantly affect the predicted dynamics. Thus, our minimal model that captures the observed dynamics is given by:

$$
\begin{aligned}
([\text{mDYT1}])' &= \alpha_{mD}(t) - \mu_{mD}[\text{mDYT1}],\\
([\text{mbHLH}])' &= \alpha_{mB}(t) - \mu_{mB}[\text{mbHLH}],\\
([\text{mTDF1}])' &= \alpha_{mT}\frac{[\text{DYT1:bHLH}]}{K_T + [\text{DYT1:bHLH}] + [\text{pMS1}]\zeta_{TM}} - \mu_{mT}[\text{mTDF1}],\\
([\text{mAMS}])' &= \alpha_{mA}\frac{[\text{pTDF1}]}{K_A + [\text{pTDF1}]} - \mu_A[\text{mAMS}],\\
([\text{mMYB80}])' &= \alpha_{mY}\frac{[\text{AMS:bHLH}]}{K_Y + [\text{AMS:bHLH}]} - \mu_Y[\text{mMYB80}],\\
([\text{mMS1}])' &= \alpha_{mM}\frac{[\text{pMYB80}]}{K_M + [\text{pMYB80}]} - \mu_M[\text{mMS1}],\\
([\text{pDYT1}])' &= \alpha_{pD}[\text{mDYT1}] - \mu_{pD}[\text{pDYT1}] - \alpha_{DB}[\text{pDYT1}][\text{pbHLH}] + \mu_{DB}[\text{DYT1:bHLH}],\\
([\text{pbHLH}])' &= \alpha_{pB}[\text{mbHLH}] - \mu_{pB}[\text{pbHLH}] - \alpha_{AB}[\text{pAMS}][\text{pbHLH}] + \mu_{AB}[\text{AMS:bHLH}]\\
&\quad - \alpha_{DB}[\text{pDYT1}][\text{pbHLH}] + \mu_{DB}[\text{DYT1:bHLH}],\\
([\text{pTDF1}])' &= \alpha_{pT}[\text{mTDF1}] - \mu_{pT}[\text{pTDF1}],\\
([\text{pAMS}])' &= \alpha_{pA}[\text{mAMS}] - \mu_{pA}[\text{pAMS}]\left(1 + \frac{\zeta_{pA}[\text{pMS1}]}{K_{pA} + [\text{pMS1}]}\right)\\
&\quad - \alpha_{AB}[\text{pbHLH}][\text{pAMS}] + \mu_{AB}[\text{AMS:bHLH}],\\
([\text{pMYB80}])' &= \alpha_{pY}[\text{mMYB80}] - \mu_{pY}[\text{pMYB80}],\\
([\text{pMS1}])' &= \alpha_{pM}[\text{mMS1}] - \mu_{pM}[\text{pMS1}],\\
([\text{AMS:bHLH}])' &= \alpha_{AB}[\text{pAMS}][\text{pbHLH}] - \mu_{AB}[\text{AMS:bHLH}],\\
([\text{DYT1:bHLH}])' &= \alpha_{DB}[\text{pDYT1}][\text{pbHLH}] - \mu_{DB}[\text{DYT1:bHLH}].
\end{aligned}
$$

This model has a marginal decrease in f^* and a smaller corrected Akaike Information Criterion (AICc, see Table II), as it has three fewer parameters, and so we give this as a preferred model to Model III. This model still has MS1 negatively regulating TDF1, as well as enhancing AMS protein degradation; the inclusion of these two feedbacks were essential to fit to the experimental data.

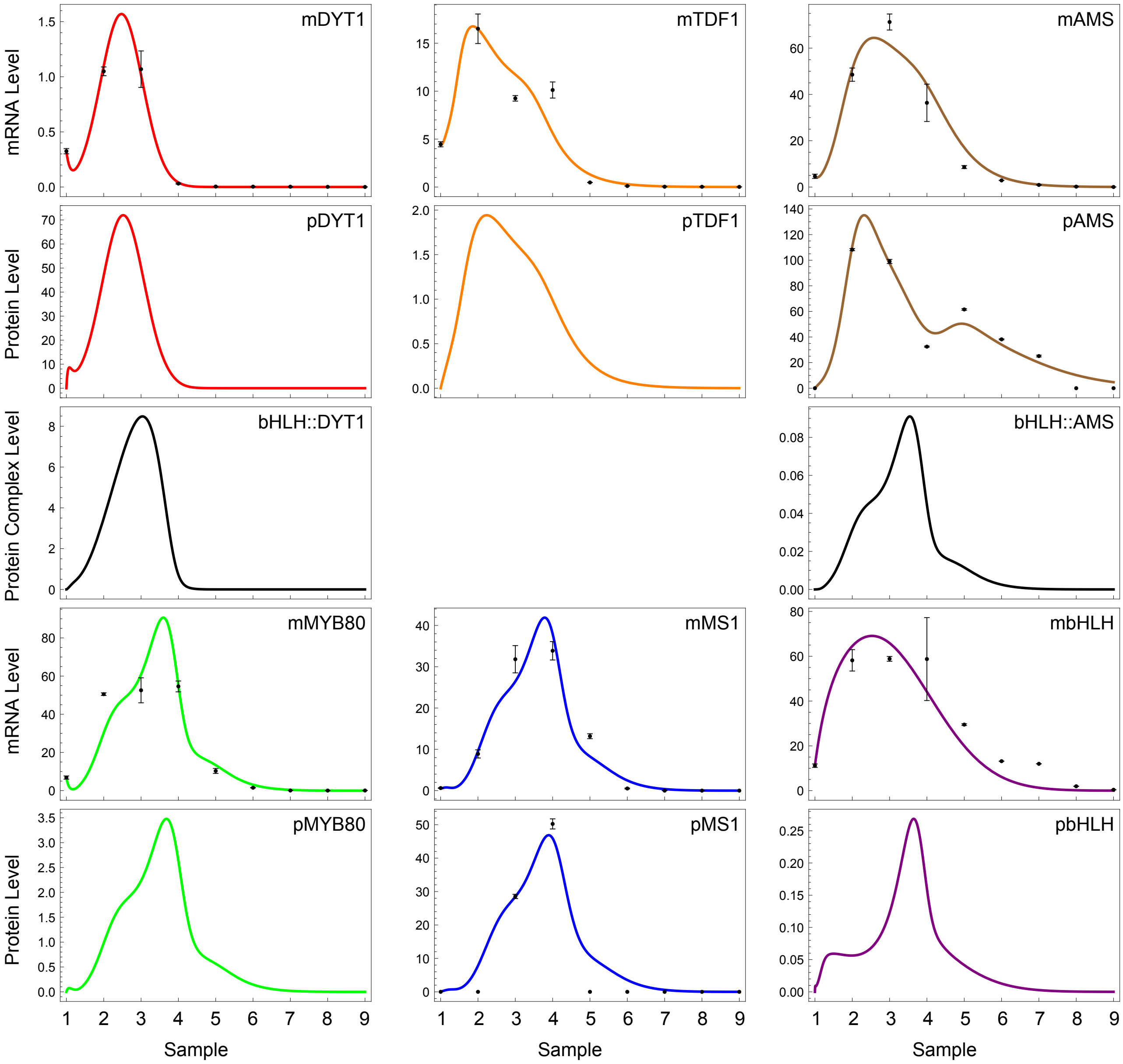

Figure V: Predicted (lines) and measured (dots with standard error bars) protein and mRNA levels for Model IV, using the model parameter values given in Table I.

5 Model V

Using the fourth model, we then added two additional potential negative feedbacks of AMS and/or MS1 inhibiting DYT1, although we are aware that the use of an exponential function for the promotion of DYT1 may mask the effect of these feedbacks.

We also note that due to the lack of data on the protein levels except AMS and MS1, these levels are entirely arbitrary, and hence we now set $\alpha_{pT} = \alpha_{pY} = \alpha_{pD} = 1$, reducing the number of parameters in the model. We also swapped to using the principal axis method (Brent, 2002) implemented in Mathematica for minimization of the objective function, this seems to optimize the fit better than the genetic algorithm in Matlab. The equations describing this model are therefore given by:

$$
\begin{aligned}
([\text{mDYT1}])' &= \alpha_{mD}(t)\frac{1}{1+\zeta_{DA}[\text{pAMS}]+\zeta_{DM}[\text{pMS1}]} - \mu_{mD}[\text{mDYT1}],\\
([\text{mbHLH}])' &= \alpha_{mB}(t) - \mu_{mB}[\text{mbHLH}],\\
([\text{mTDF1}])' &= \alpha_{mT}\frac{[\text{DYT1:bHLH}]}{K_T+[\text{DYT1:bHLH}]+[\text{pMS1}]\zeta_{TM}} - \mu_{mT}[\text{mTDF1}],\\
([\text{mAMS}])' &= \alpha_{mA}\frac{[\text{pTDF1}]}{K_A+[\text{pTDF1}]} - \mu_A[\text{mAMS}],\\
([\text{mMYB80}])' &= \alpha_{mY}\frac{[\text{AMS:bHLH}]}{K_Y+[\text{AMS:bHLH}]} - \mu_Y[\text{mMYB80}],\\
([\text{mMS1}])' &= \alpha_{mM}\frac{[\text{pMYB80}]}{K_M+[\text{pMYB80}]} - \mu_M[\text{mMS1}],\\
([\text{pDYT1}])' &= \alpha_{pD}[\text{mDYT1}] - \mu_{pD}[\text{pDYT1}] - \alpha_{DB}[\text{pDYT1}][\text{pbHLH}] + \mu_{DB}[\text{DYT1:bHLH}],\\
([\text{pbHLH}])' &= \alpha_{pB}[\text{mbHLH}] - \mu_{pB}[\text{pbHLH}] - \alpha_{AB}[\text{pAMS}][\text{pbHLH}] + \mu_{AB}[\text{AMS:bHLH}]\\
&\quad - \alpha_{DB}[\text{pDYT1}][\text{pbHLH}] + \mu_{DB}[\text{DYT1:bHLH}],\\
([\text{pTDF1}])' &= \alpha_{pT}[\text{mTDF1}] - \mu_{pT}[\text{pTDF1}],\\
([\text{pAMS}])' &= \alpha_{pA}[\text{mAMS}] - \mu_{pA}[\text{pAMS}]\left(1+\frac{\zeta_{pA}[\text{pMS1}]}{K_{pA}+[\text{pMS1}]}\right)\\
&\quad - \alpha_{AB}[\text{pbHLH}][\text{pAMS}] + \mu_{AB}[\text{AMS:bHLH}],\\
([\text{pMYB80}])' &= \alpha_{pY}[\text{mMYB80}] - \mu_{pY}[\text{pMYB80}],\\
([\text{pMS1}])' &= \alpha_{pM}[\text{mMS1}] - \mu_{pM}[\text{pMS1}],\\
([\text{AMS:bHLH}])' &= \alpha_{AB}[\text{pAMS}][\text{pbHLH}] - \mu_{AB}[\text{AMS:bHLH}],\\
([\text{DYT1:bHLH}])' &= \alpha_{DB}[\text{pDYT1}][\text{pbHLH}] - \mu_{DB}[\text{DYT1:bHLH}].
\end{aligned}
$$

The final result, as shown in Figure VI, does includes the negative feedback of DYT1 by AMS, but not by MS1 (and we therefore set $\zeta_{DM} = 0$). This model was also tested for the inclusion of the three negative feedbacks removed in model IV, this did not change the conclusion that these should be removed.

Using the HYPERSPACE algorithm again, as shown in Figure VII, gives $9,168$ parameters sets within 10% of the best f^* value found. This shows that the majority of the parameters are well-constrained around the values in Table I, except for the constant b_B from the exponential function which drives bHLH expression, which is essentially zero and could be removed from the model.

Our final model therefore includes AMS inhibiting DYT1 and MS1 inhibiting TDF1, as well as MS1 increasing the degradation rate of AMS protein.

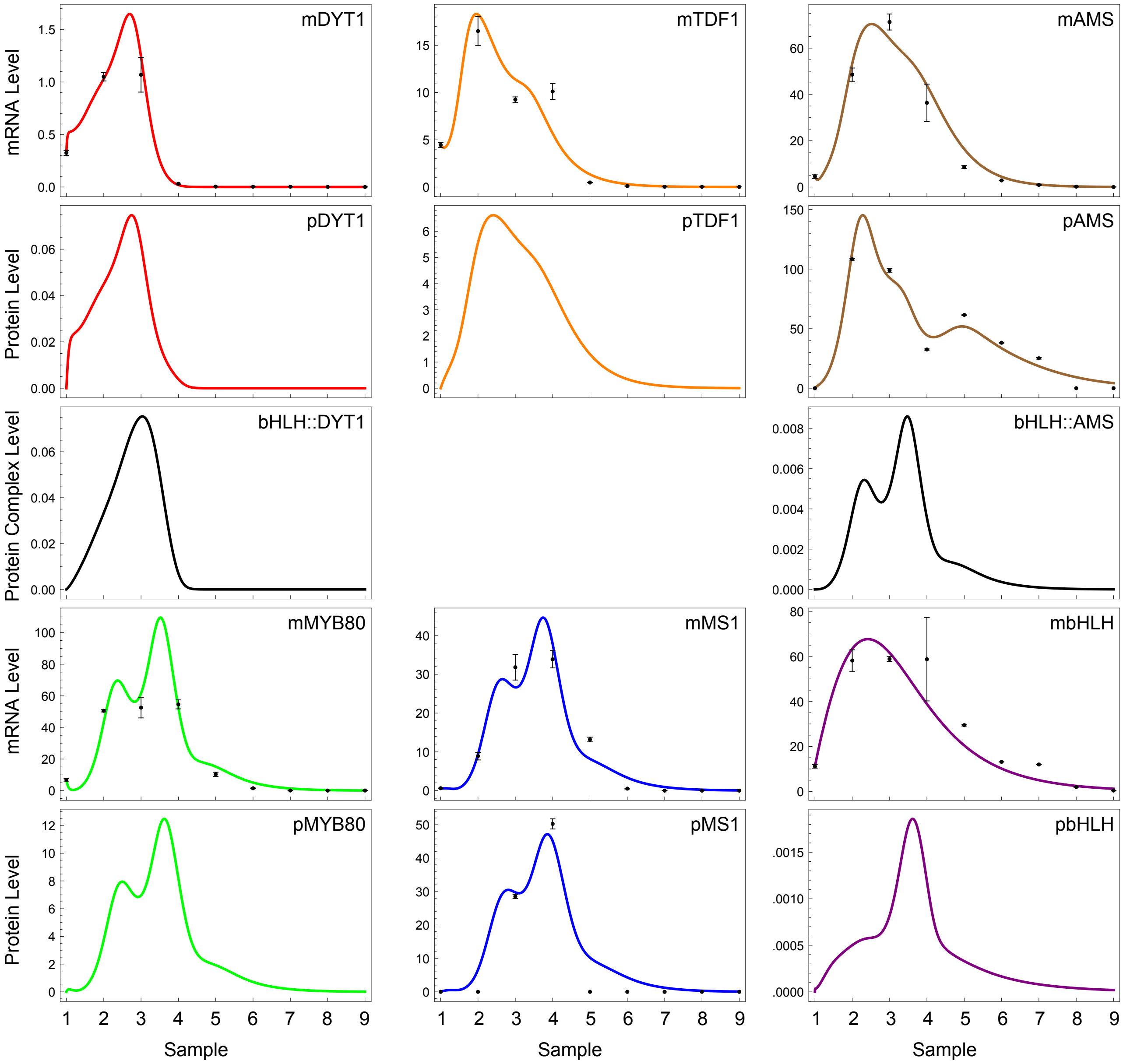

Figure VI: Predicted (lines) and measured (dots with standard error bars) protein and mRNA levels for Model V, using the model parameter values given in Table I.

	Model I	Model II	Model III	Model IV	Model V
a_{mD}	1.341	1.0729	1.0336	1.2182	3.5685
b_D	0.16005	0.1414	0.1251	0.1403	0.1616
c_D	-0.02336	-0.0431	-0.1491	-0.1054	-0.2089
μ_{mD}	1.1962	0.9141	0.7922	1.0162	1.0635
α_{mT}	3.7589	3.0332	2.5664	2.4742	3.4919
K_{mT}	1.284	0.7030	-0.2194	-0.6609	-0.1856
μ_{mT}	0.8930	0.8548	0.2291	0.1706	0.1665
α_{mA}	4.5304	2.9137	3.6552	3.2129	4.2118
K_{mA}	1.6435	0.9106	1.5624	1.0322	3.9653
μ_{mA}	0.4261	0.42347	0.5200	0.5673	1.0174
α_{mY}	4.9516	3.0006	4.4816	4.2121	5.3342
K_{mY}	4.5781	1.7227	0.0114	-0.0556	-0.0964
μ_{mY}	0.6575	0.5306	1.2145	1.2237	1.317
α_{mM}	4.9368	4.6266	4.2151	3.6899	3.9932
K_{mM}	3.1689	3.009	2.2539	1.6915	2.5715
μ_{mM}	0.5020	0.5484	1.0177	0.8769	0.834
a_{mB}	n/a	3.2008	2.3934	2.3317	1.9571
b_B	n/a	0.2544	0.0814	0.0702	-1.5331
c_B	n/a	0.2762	0.3731	0.3406	0.2405
μ_{mB}	n/a	1.3529	0.5530	0.4811	-0.1501
α_{pD}	0.4452	1.7872	3.4344	3.0000	0
μ_{pD}	0.2156	-0.1724	1.3369	1.3355	1.3485
α_{pT}	-0.8529	0.1092	-0.40406	-0.3645	0
μ_{pT}	0.7451	0.7175	0.2733	0.5308	0.3403
α_{pA}	0.9393	1.0249	0.94953	0.8907	0.903
μ_{pA}	-0.1123	0.3939	0.2547	-0.0442	-0.0401
α_{pY}	-1.0099	-0.7430	-0.0677	-0.4009	0
μ_{pY}	0.50111	0.5494	1.1799	1.0074	0.9407
α_{pM}	0.65051	0.7288	0.8569	0.9379	0.9141
μ_{pM}	0.60883	0.6843	0.8176	0.879	0.8751
α_{pB}	n/a	0.7744	-0.8847	-0.7863	-2.7403
μ_{pB}	n/a	3.4935	1.9287	1.9039	1.9577
ζ_{TM}	0.56579	0.5649	0.7117	0.7762	-0.2696
ζ_{TA}	-3.071	-2.6597	-1.8183	n/a	n/a
ζ_{MM}	-2.4872	-2.0236	-1.6413	n/a	n/a
ζ_{AM}	0.0956	-0.5933	0.0607	n/a	n/a
ζ_{pAM}	1.4913	3.095	3.0258	2.681	3.7257
K_{pA}	1.1946	1.9352	1.996	1.9582	3.7647
α_{AB}	n/a	n/a	-0.88059	-0.8797	1.1176

μ_{AB}	n/a	n/a	1.4447	1.3934	2.2964
α_{DB}	n/a	n/a	1.5156	1.7552	5.8060
μ_{DB}	n/a	n/a	1.6964	1.6173	2.6633
ζ_{DA}	n/a	n/a	n/a	n/a	0.1480

Table I: Best-fit $\log_{10}$ values of the parameters in the five different models; n/a denotes a parameter not included in that model.

	Model I	Model II	Model III	Model IV	Model V
f^*	1.6734	1.3869	0.8129	0.7913	0.6994
n_{pars}	32	38	42	39	37
n_{data}	63	72	72	72	72
AICc	-322.763	-402.924	-497.555	-474.102	-512.690

Table II: Values of the objective function, number of parameters, number of datapoints and corrected Akaike Information Criterion (AICc) for the best fit found for the five models. Smaller values of f^* and $AICc = 2 * n_{pars} * n_{data}/(n_{data} - n_{pars} - 1) + 2n_{data} \ln(f^*/n_{data})$ represent a better fit to the data.

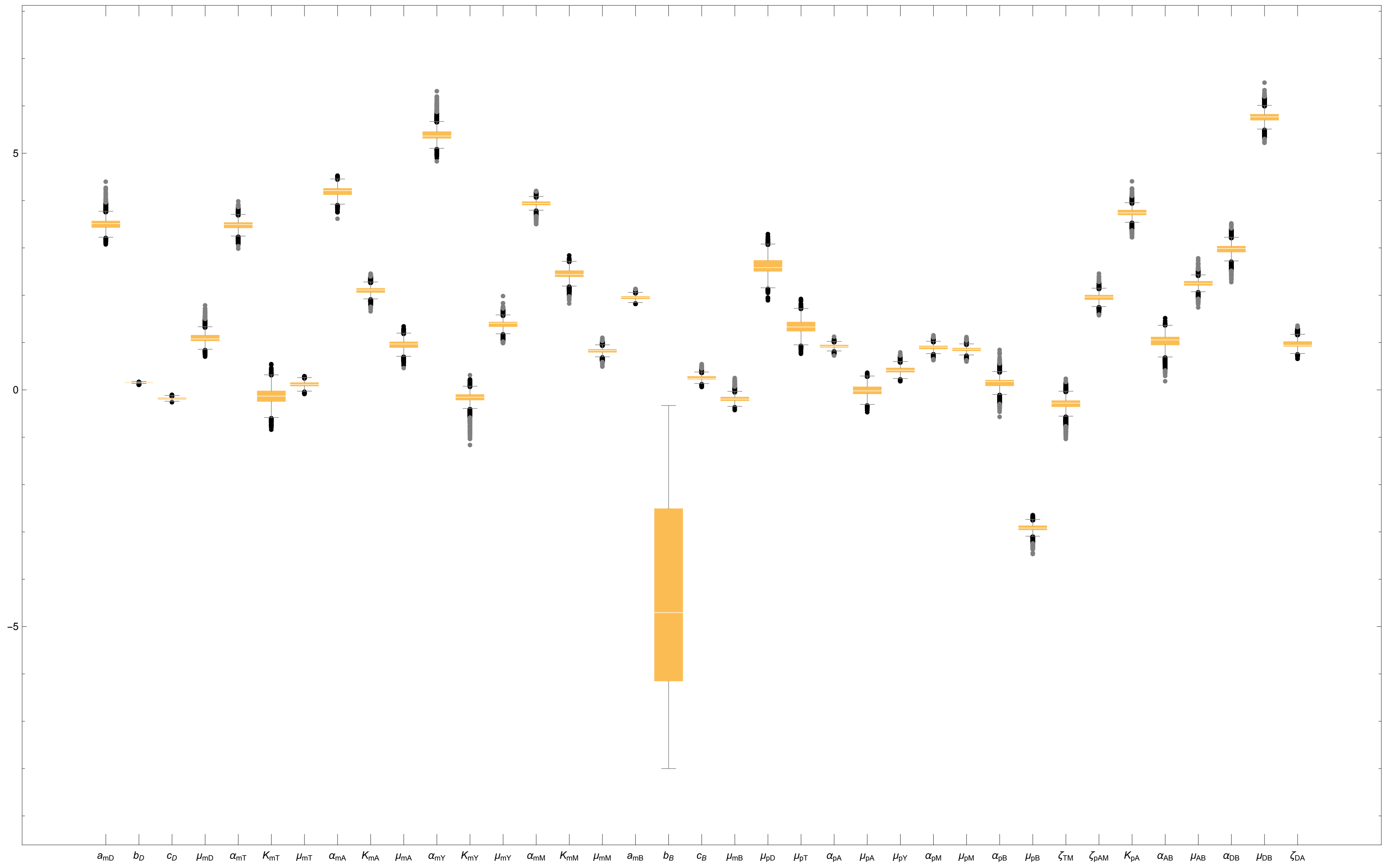

Figure VII: Set of boxplots from running the HYPERSPACE algorithm, showing the variability of the 37 parameters in Model V, for parameter combinations that achieve a similarly good fit as the set of values in Table I

.

6 Simulation of Mutant Phenotypes

We now simulate the effect of different mutants from Model V. First, we consider the *ms1* mutant in Figure VIII, where we block the production of MS1 protein and mRNA, as well as setting the initial mRNA level to be zero. In this simulation, the lack of MS1 causes an up-regulation of TDF1 and AMS, as is seen in the mutant data (Figure S4), and the AMS protein is tied to the AMS mRNA levels.

The *ams* mutant is shown in Figure IX, where we block the production of AMS protein and mRNA, $\alpha_{mA} = \alpha_{pA} = 0$, and set the initial mRNA level of AMS to be zero. This has the effect of an increase of TDF1, as the downstream genes (MYB80 and MS1) are not present to cause TDF1's downregulation via MS1.

We also show in Figure X the effect of removing the enhanced AMS protein degradation by MS1, $\zeta_{pAM} = 0$, it can be seen that the AMS protein levels then remain coupled to the AMS mRNA levels, as would be expected.

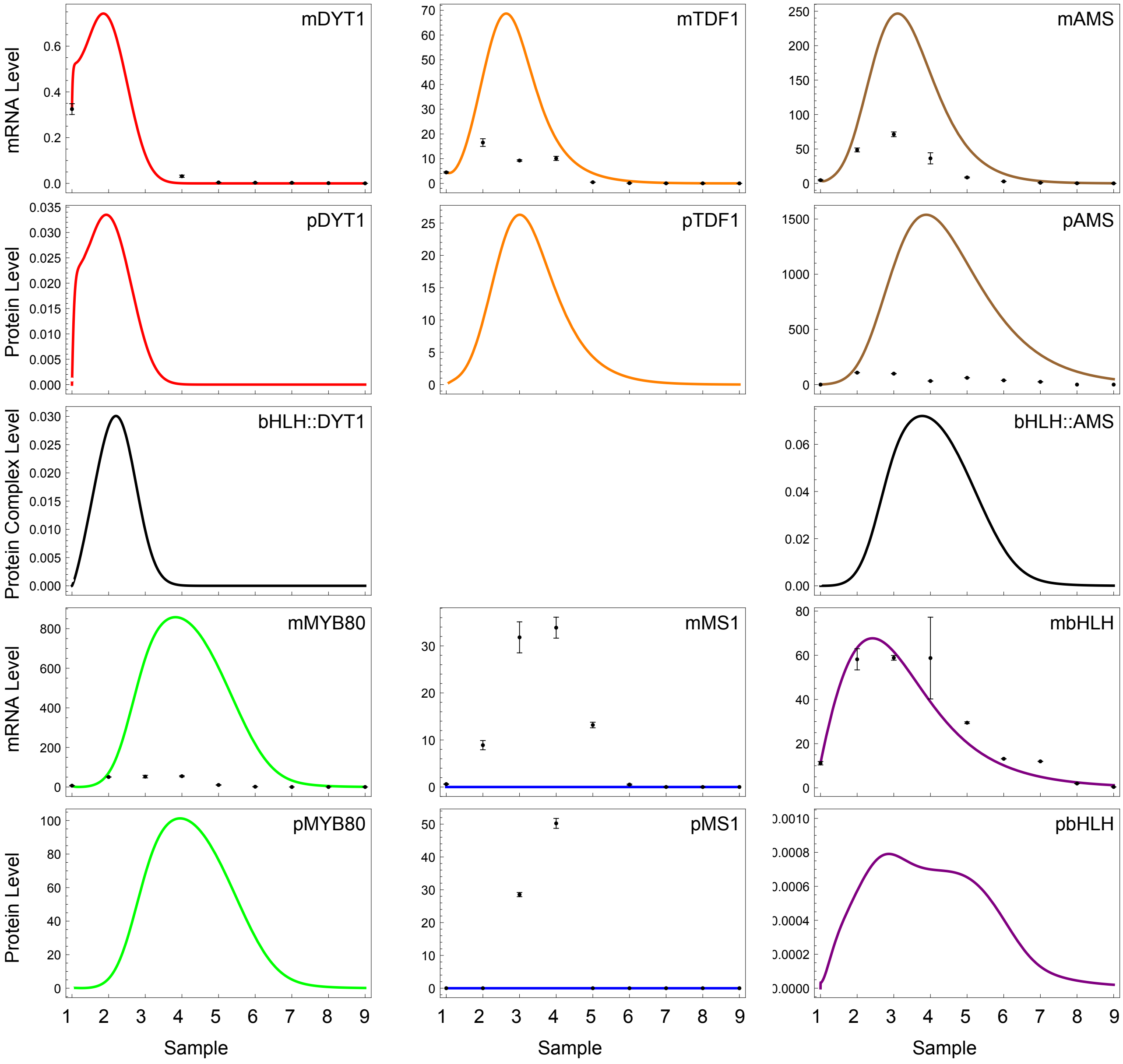

Figure VIII: Predicted (lines) and measured (dots with standard error bars) protein and mRNA levels for the *ms1* mutant, $\alpha_{mM} = \alpha_{pM} = 0$, using the model parameter values given for Model V in Table I.

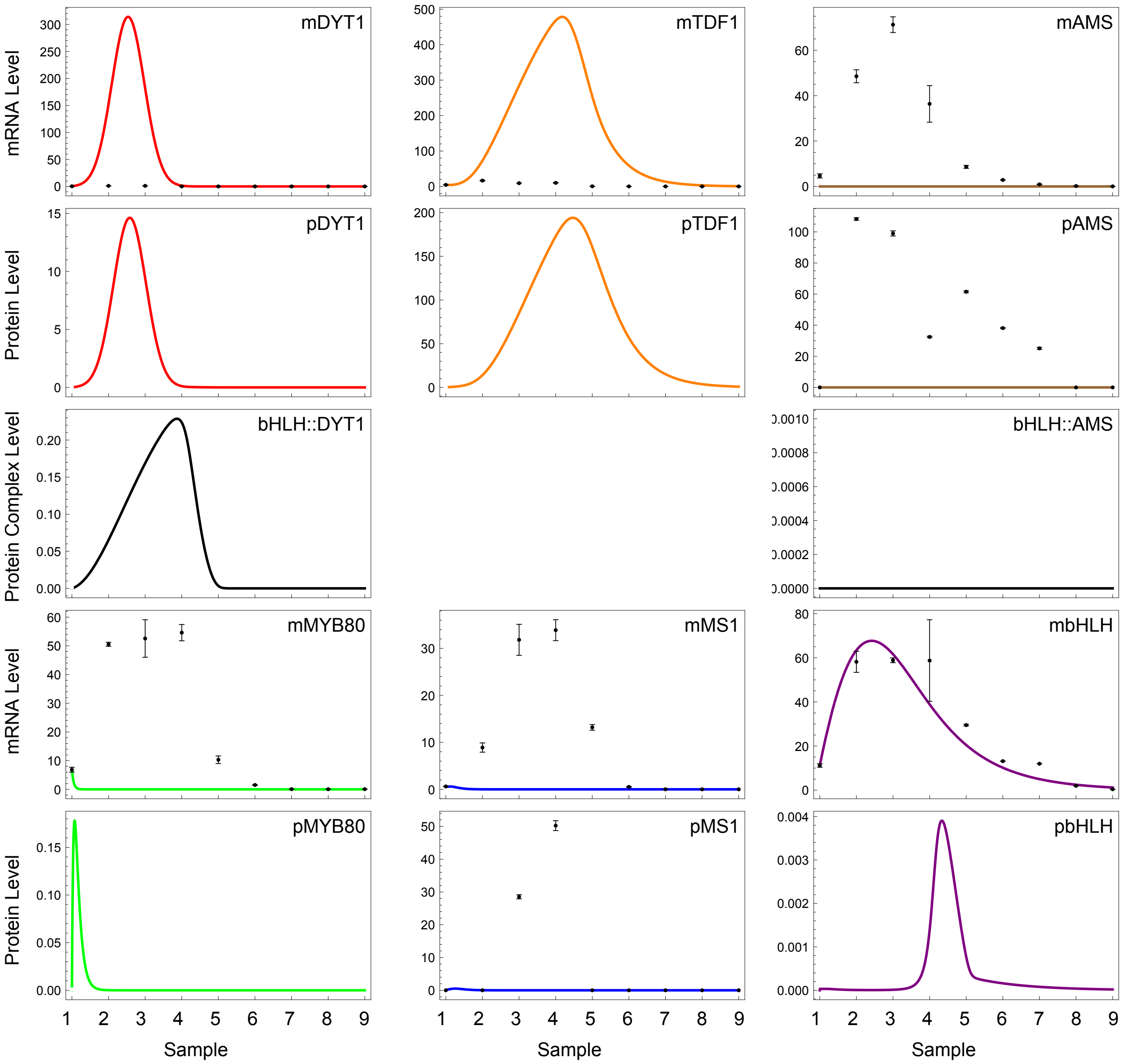

Figure IX: Predicted (lines) and measured (dots with standard error bars) protein and mRNA levels for the *ams* mutant, $\alpha_{mA} = \alpha_{pA} = 0$, other parameter values as given for Model V in Table I.

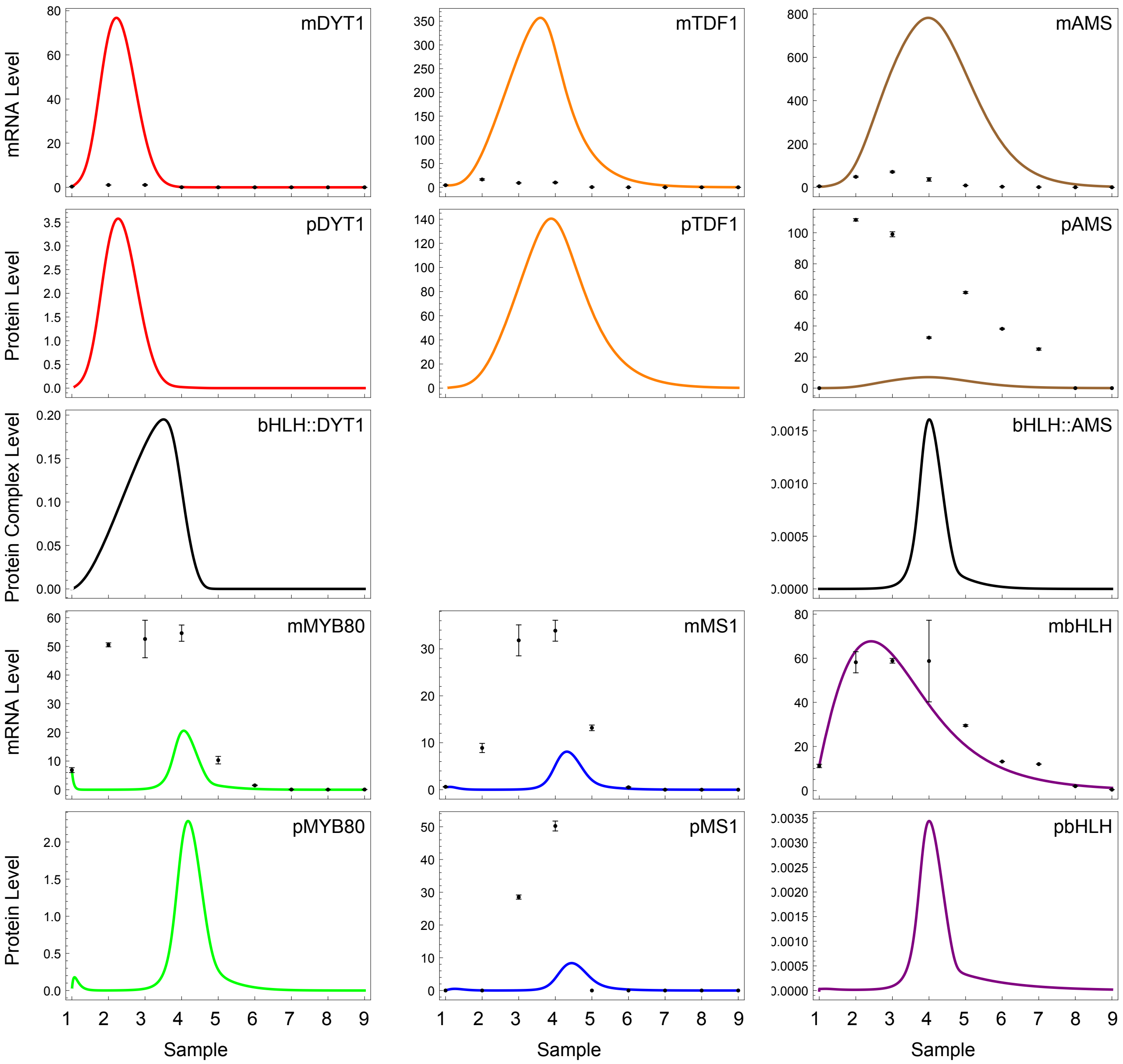

Figure X: Predicted (lines) and measured (dots with standard error bars) protein and mRNA levels for the removal of MS1 enhancing AMS protein degradation in Model V, $\zeta_{pAM} = 0$, other parameter values as given in Table I.

References

Brent, R. P. (2002). *Algorithms for minimization without derivatives*. New York: Dover Publications.

Feng, B., D. Lu, X. Ma, Y. Peng, Y. Sun, G. Ning, and H. Ma (2012). Regulation of the arabidopsis anther transcriptome by dyt1 for pollen development. *The Plant Journal 72*(4), 612–624.

Xu, J., C. Yang, Z. Yuan, D. Zhang, M. Y. Gondwe, Z. Ding, W. Liang, D. Zhang, and Z. A. Wilson (2010). The aborted microspores regulatory network is required for postmeiotic male reproductive development in arabidopsis thaliana. *The Plant Cell 22*(1), 91–107.

Zamora-Sillero, E., M. Hafner, A. Ibig, J. Stelling, and A. Wagner (2011). Efficient characterization of high-dimensional parameter spaces for systems biology. *BMC Systems Biology 5*(1), 142.
